# microRNA blood signature for localized radiation injury

**DOI:** 10.1038/s41598-024-52258-2

**Published:** 2024-02-01

**Authors:** Lucie Ancel, Olivier Gabillot, Chloé Szurewsky, Romain Granger, Amandine Sache, Frédéric Voyer, Gaëtan Gruel, Stéphane Illiano, Marc Benderitter, Bernard Le Guen, Maâmar Souidi, Mohamed Amine Benadjaoud, Stéphane Flamant

**Affiliations:** 1 Radiobiology and Regenerative Medicine Research Service, Radiobiology of Accidental Exposure Laboratory, IRSN PSE-SANTE/SERAMED/LRAcc, 31 av de la Division Leclerc, 92260 Fontenay-aux-Roses, France; 2 Ionizing Radiations Biological and Sanitary Effects Research Service, Support Group for Research and Animal Ethic, IRSN PSE-SANTE, Fontenay-aux-Roses, France; 3 Radiobiology and Regenerative Medicine Research Service, IRSN PSE-SANTE, Fontenay-aux-Roses, France; 4 EDF, DPN, Saint-Denis, France

**Keywords:** Non-coding RNAs, Diagnostic markers

## Abstract

A radiological accident, whether from industrial, medical, or malicious origin, may result in localized exposure to high doses of ionizing radiations, leading to the development of local radiation injury (LRI), that may evolve toward deep ulceration and necrosis of the skin and underlying tissues. Early diagnosis is therefore crucial to facilitate identification and management of LRI victims. Circulating microRNAs (miRNA) have been studied as potential diagnostic biomarkers of several diseases including hematological defects following whole-body irradiation (WBI). This study aims to identify a blood miRNA signature associated with LRI in a preclinical C57BL/6J mouse model of hindlimb irradiation using different 10-MV X-ray doses that lead to injuries of different severities. To this end, we first performed broad-spectrum plasma miRNA profiling, followed by a targeted validation step, on two independent animal cohorts. Using a multivariate sparse partial least square discriminant analysis, we identified a panel of eight circulating miRNAs able to segregate mice according to LRI severity. Interestingly, these miRNAs were previously associated with WBI (miR-150-5p, miR-342-3p, miR-146a-5p), inflammation (miR-18a-5p, miR-148b-3p, miR-532-5p) and skin diseases (miR-139-5p, miR-195-5p). Our results suggest the use of circulating miRNAs as suitable molecular biomarkers for LRI prognosis and diagnosis.

## Introduction

In the context of a radiological accident of medical or industrial origin, or a malicious act, victims exposed to high doses of ionizing radiation in a suspected or proven manner may develop radiation induced injuries. Whole-body irradiation (WBI) can lead to the development of an acute radiation syndrome (ARS), with hematopoietic, gastrointestinal or neurovascular manifestations, depending on several parameters such as radiation dose, duration of exposure and volume of irradiated body^1^. In the case where radiation exposure is restricted to a limited area of the body, a local radiation injury (LRI) may occur, which results in alterations to the skin and underlying tissues including muscle, bones and vasculature for high doses^2,3^.

LRI is a dynamic lesion characterized by an asymptomatic latency phase immediately after exposure, followed by a manifestation phase characterized by erythema, dry or moist desquamation and ulceration for highest doses (> 20 Gy), evolving in a succession of unpredictable inflammatory waves^4–7^. Indeed, as clinical manifestations are dose and time-dependent, fibrosing inflammatory flares and necrotic recurrences may occur months or years after exposure for the most serious cases^8,9^. Thus, healing of LRI is a long process where pain induced by the injury remains resistant to opiates^10^. Access to a tool for early diagnosis of LRI would be therefore particularly useful for the management of a radiological emergency consecutive to mass accident. Such early identification of potential victims of severe LRI would improve medical management with regard to victim surveillance, appropriate assignment to ad hoc hospitals, and patient preparation for adapted therapeutic procedures^3^. However, no molecular marker is currently available to prognosticate the appearance and/or the severity of LRI before emergence of the first symptoms.

For a clinical use in a case of emergency, interesting biomarkers should be stable, collected with a minimally invasive method (a simple blood test for example) and analyzed easily at a reasonable cost^10^. In this regard, circulating microRNAs (miRNAs) constitute promising candidates as biomarkers of radiation induced biological effects. These tiny, non-coding RNA molecules of 18–22 nucleotides long, regulate gene expression by silencing or destabilizing targeted messenger RNAs^11^. Their level of expression can vary according to various stresses or environmental conditions^12^. Their interest as diagnostic, predictive or therapeutic biomarkers has been demonstrated in a number of human pathologies, notably in different types of cancer^13,14^ or cardiovascular diseases^15^. MiRNAs are both present in tissues and blood circulation, and changes in circulating miRNAs have already been correlated with specific tissue damage. For example, Fang et al. identified an 8-miRNA plasmatic signature associated with myocardial fibrosis in human^16^, while Ichihara et al. described miRNAs as key regulators and useful serum biomarkers of severe cutaneous injury resulting from drug toxicity^17,18^. Interestingly, the relevance of blood miRNAs as biomarkers of WBI has been demonstrated in mice and in non-human primates (NHPs)^19,20^. A recent study on primates demonstrated a specific signature of circulating miRNAs for whole thorax lung irradiation compared to WBI, which confirms the potential of miRNA blood signatures for clinical use in the context of irradiation^21^. Similarly, an early signature of serum miRNAs indicating long-term impact on the hematopoietic system was identified after WBI in mice^22^. These results suggest that circulating miRNAs can be used for an early assessment of radiological damages and for the management of long-term radiation impact. However, the interest of blood miRNAs as molecular markers for diagnosis of acute LRI has not been documented.

With the objective to establish early miRNA-based signatures predictive of the risk of developing LRI, we first performed a proof-of-principle study that aimed at demonstrating the relevance of using systemic miRNA blood measurements as LRI biomarkers. Here we describe the first molecular signature of circulating miRNAs associated with LRI, using a preclinical mouse model of local irradiation at different doses of X-rays. We analyzed the composition of miRNAs in blood and studied their correlation with the injury score and several clinical parameters, such as complete blood count (CBC), plasmatic C-reactive protein (CRP) levels, and cutaneous blood perfusion.

## Materials and methods

### Experimental model

#### Animals

Experiments were conducted in compliance with French veterinary guidelines and those formulated by the European Community for experimental animal use, protocols were approved by the institutional animal experimentation and ethics committee. A total of 120 male 8-week-old C57BL/6 mice (Janvier Labs, Le Genest Sainte Isle, France) were used in this study. Animals were housed in cages in temperature and humidity-controlled chamber, with alternating 12 h light and dark cycles and *ad lib* access to water and food. Mice were acclimated for 1 week upon arrival, before undergoing experimental procedure.

#### Study design

The study was conducted in 2 steps, using 2 independent animal cohorts. First, 60 mice (15/group) were used for the broad-spectrum screening step that allowed to identify a preliminary miRNA signature that could discriminate individuals according to their dose group and the severity of the injury. This signature was then challenged using an independent cohort of another 60 animals (15/group), in which we measured the plasma levels of selected miRNAs, which resulted in the final miRNA signature.

#### Irradiation and experimental procedure

**Figure 1:**
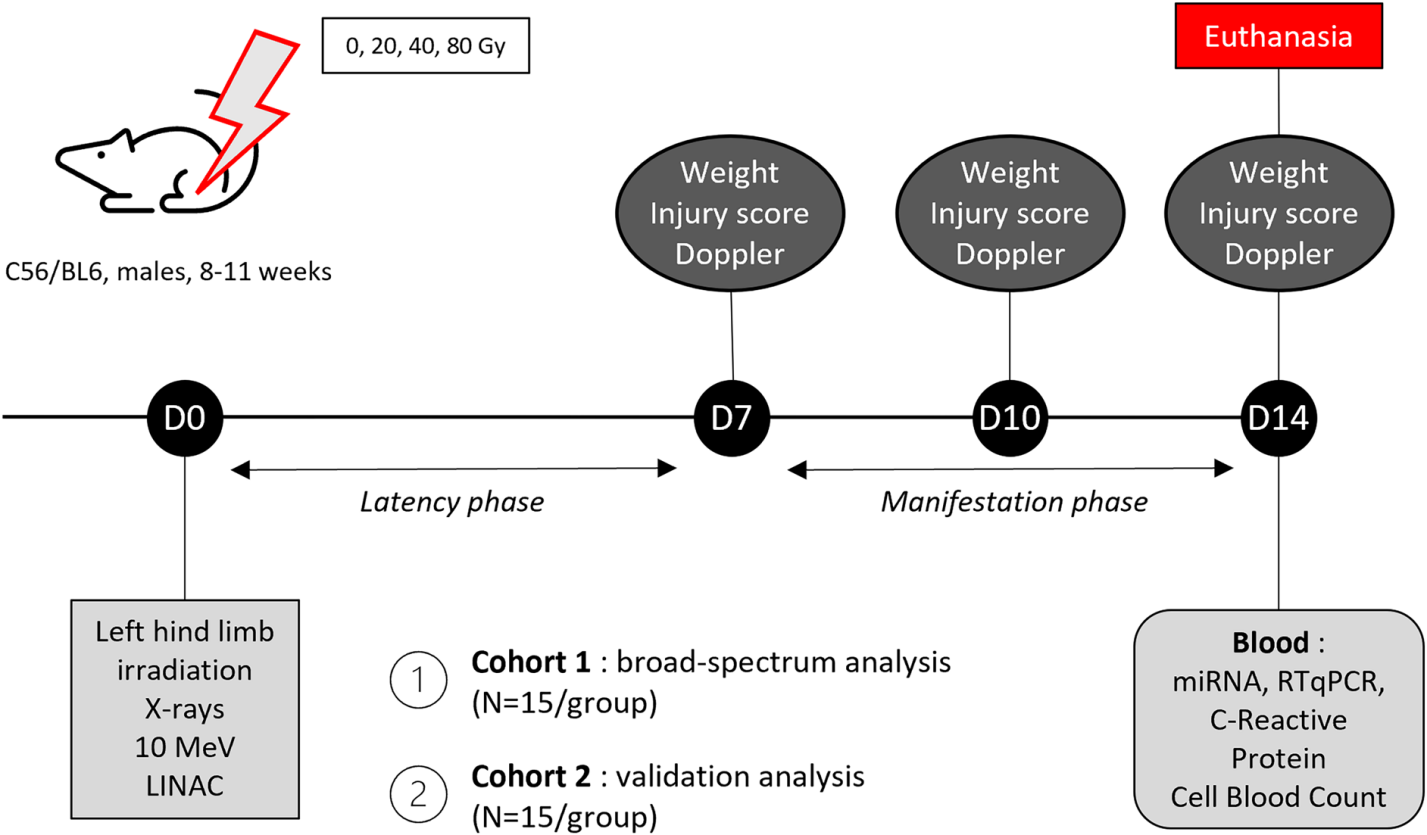
Experimental design. Two independent mice cohort were used with the same experimental design, to perform a broad-spectrum profiling of miRNAs (cohort 1) and then a targeted study (cohort 2). For each cohort, mice were irradiated according to 4 irradiation groups with 20, 40 or 80 Gy and 0 for the control group (N = 15/groups) with 10 MV X-rays with a LINAC at a dose rate of 2.5 Gy/min under anesthesia performed with isoflurane (D0). Injury score and blood perfusion analysis were performed at D7, D10 and D14. Mice were euthanized at D14 after blood samplings.

After hair removing on both hindlimbs, mice were irradiated on the left hind limb, foot excluded, with X-rays at 10 MV using a medical linear accelerator (Elekta SAS, Boulogne-Billancourt, France) at a dose rate of 3 Gy/min under anesthesia performed with isoflurane. Dose rate measurement was performed with an ionization chamber (PTW 31,010–0125 cc) in Kair on a plexiglass plate (uncertainty of 5% at k = 2). After being randomly split in four different dose groups, mice were irradiated 5 at a time with a single dose of 20, 40 or 80 Gy and 0 Gy for control group (N = 15/group) (Fig. 1). The field size was a rectangle of 2 × 30 cm, and the distance source-sample was 1 m. During the experiment, 5 mice from the 40 Gy group have been excluded from the study because of aggressive behavior between littermates. Every manipulation handling and non-invasive measurements on animals were performed under anesthesia with isoflurane. Mice were euthanized fourteen days after irradiation with intracardiac blood sampling followed by cervical dislocation.

This whole procedure was replicated on a second independent cohort (N = 15 animals/group) for validation of miRNA signature in the same conditions.

### Sample collection

At D14 (end of experiment), when the injury is well-established, 100 μL of retro-orbital blood was collected with micropipettes and transferred to a BD Microtainer K2 EDTA tube (BD Biosciences, Le Pont de Claix, France) for CBC analysis.

Around 1 mL of intracardiac blood was collected using a syringe pre-coated with 0.105 M sodium citrate and transferred in a 1.5 mL tube containing 75 μL of 0.105 M citrate before processing to obtain plasma. The sample was centrifuged at 1500×*g* for 15 min at 4 °C. The supernatant was collected and processed to obtain platelet-poor plasma (PPP), with a second centrifugation at 13,000×*g* for 2 min at 4 °C. The PPP was then stored at  −80 °C until use.

### Clinical approach

#### Injury score

Injury score was independently established by two experimenters according to a grid of clinical criteria partially inspired by the METREPOL system^23^, including erythema, lesion extent, oedema, desquamation, wound moisture and limb retraction (supplementary Fig. 1). Each criterion was graded from 0 to 1 and summed to generate a global injury score as previously described^24^. The mean of the two experts’ observational scores was computed and used for the analysis (Supplementary Table 1). Of note, this preclinical injury score was essentially designed to estimate the severity of injuries, and not to define treatment strategies.

#### Blood cutaneous perfusion

Skin blood perfusion imaging of the irradiated and contralateral limbs was performed using a Moor LDI2 Laser Doppler Imager (Moor Instruments ltd, Axminster, UK). During the measure (5 min), mice were under isoflurane and placed on a heating plate at 37 °C to minimize temperature variation. Blood perfusion was expressed as the irradiated/non-irradiated limbs ratio.

#### Weight

Animal weigh was monitored at days 0, 7 and 14.

### Molecular approach

#### MiRNA extraction and quality control

RNAs were extracted from blood sample with the miRNeasy® Plasma/Serum Advanced kit (Qiagen, Courtaboeuf, France) according to manufacturer’s instructions. A quality control of the extraction product was performed with a qPCR analysis using three different miRNAs assays as controls: one with ubiquitous expression (miR-23a-3p), another reflecting potential hemolysis (miR-451a) and finally an exogenous miRNA added in the sample just before the extraction (cel-miR-39) to control the extraction process. The degree of hemolysis of each sample was determined by computing the difference between miR-23a-3p and miR-451a expression levels, as previously described^25^. All samples that did not pass the quality control were excluded from the study.

#### Broad-spectrum analysis

Reverse transcription was performed with the TaqMan™ microRNA RT kit and rodent Megaplex RT primer pools set v3.0 (Fisher Scientific, Illkirch, France) in 7.5 µL reaction volume, following manufacturer’s instructions, on a Mastercycler® X50 (Eppendorf, Hamburg, Germany). The resulting cDNAs (2.5 µL) were pre-amplified (12 cycles) using Megaplex PreAmp primers and TaqMan PreAmp mastermix (Fisher Scientific) in a final volume of 25 µL, on the Mastercycler X50 (Eppendorf). The PreAmp products were then diluted to 100 µL with TE buffer, and 9 µL of diluted PreAmp products were used to prepare 900 µL of PCR reaction mix with TaqMan Universal Mastermix II, no UNG (Fisher Scientific), to load each of the 2 TaqMan low density array (TLDA) Rodent MicroRNA A + B Cards Set v3.0 (Fisher Scientific). Quantitative (q) PCR was run on an ABI 7900HT instrument (Applied Biosystems, Foster City, CA) for 40 cycles. Cycle thresholds (Ct) for each assay were determined using automatic baseline with Expression-Suite Software v1.3 (Applied Biosystems), and Ct values > 37 were discarded. Global normalization was applied to this dataset using DataAssist Software v3.01 (Applied Biosystems) to compute Delta-Ct values for subsequent multivariate analyses.

#### Targeted qPCR analysis

Reverse transcription was performed with the miRCURY® LNA RT Kit (Qiagen), following manufacturer’s procedure, using a Mastercycler® X50 (Eppendorf). QPCR was performed using the miRCURY® LNA SYBR Green PCR Kit (Qiagen), on an ABI 7900HT instrument (Applied Biosystems) in 10 µL reaction volume for 40 cycles, using 3 μL of 1:30 diluted cDNA. Delta-Ct values from selected miRNAs were computed following adaptive normalization using the ‘MiRAnorm' package in R Software^26^ and were used for multivariate analysis and generation of the validation signature.

#### CRP Levels and complete blood count

In order to reproduce the usual clinical management, we measured markers of inflammation (CRP levels) and general health (CBC) at D14 post-irradiation. Quantification of CRP was performed using Quantikine ELISA Mouse C-Reactive Protein test (R&D Systems, Abingdon, UK) according to the manufacturer’s protocol with plasma diluted at 1:4000 with provided buffer. CBC analysis at day 14 was performed on EDTA total blood using an ABX Micros ES 60 (Horiba, Longjumeau, France) according to manufacturer’s instructions.

### Statistical and multivariate analyses

Statistical analysis of data from weight monitoring, CBC, CRP levels, injury score, and cutaneous blood perfusion were performed on GraphPad Prism® v9 with a Kruskal–Wallis test (non-parametric).

A supervised multivariate analysis was conducted to model a multi-scale signature (comprising miRNA expression, CRP and CBC, weight monitoring, injury score, and cutaneous blood perfusion) able to simultaneously discriminate the different dose groups and highlight the most significant correlations between the different entities of each scale.

More precisely, we defined three scales (blocks) of covariates acquired for each sample: (1) miRNA block with all the miRNA expression measures, (2) Blood block with CRP and CBC and (3) Clinical block which includes weight monitoring, injury score, and cutaneous blood perfusion.

To conduct an integrative study by analyzing together all the blocks described above, we performed a sparse Multiblock partial least square discriminant analysis (sPLS-DA) model^27^ via the DIABLO framework of the *mixOmics* package (version 6.18.1) in R software.

DIABLO extends regularized and sparse generalized canonical correlation analysis of blocks of covariates measured in a continuous scale to a classification framework by including an additional block formed by a dummy indicator matrix to indicate the dose group membership of each sample. Thus, this model is particularly appealing in the context of our study since it permits to avoid any subjective thresholding or categorization of the injury scores (Clinical block) by keeping its values in their original continuous scale in one hand, and in the other hand by taking into account the dose groups naturally expressed as a categorical covariate.

DIABLO maximizes the covariance (correlation) between the several blocks by constructing components (linear combinations of the original covariates of each block—miRNA, Blood and Clinical) that are maximally correlated with respect to an outcome variable (in this case, the dose groups) to simultaneously performing association and classification tasks. In this study, we set a value of 0.5 in the design matrix of the model in order to achieve a balanced tradeoff between correlation and discrimination tasks^28^.

The derived multi-scale signature from this model is consequently optimal to *jointly* discriminate the dose groups and maximize the correlation between the different blocks and especially between the miRNA expression levels and the severity of radiation-induced injuries.

The lasso penalization in the loss function of the model ensures sparsity, variable selection (in particular the miRNAs selection in the miRNA block), and interpretability. In all the analyses, leave-one-out cross validation was used to determine the model parameters including number of components and number of features per component. The cross-validation is a model validation technique used to assess whether the results of an analysis can be generalized to another data set. It consists in dividing the data set into folds, training the model on all folds except one and evaluating the prediction performance on the left-out subset. This process is iterated until each subset is left out once; in the case of Leave-One-Out CV used in this study, each fold consists of one sample which is left out once.

### Ethics approval

Experiments were conducted in compliance with French veterinary guidelines and those formulated by the European Community for experimental animal use. Protocols were approved by the animal experimentation and ethics committee of IRSN (APAFIS Agreement No. 22393-2019101116207862v1) and were carried out in accordance with the ARRIVE guidelines (http://arriveguidelines.org).

## Results

### Injury monitoring

**Figure 2:**
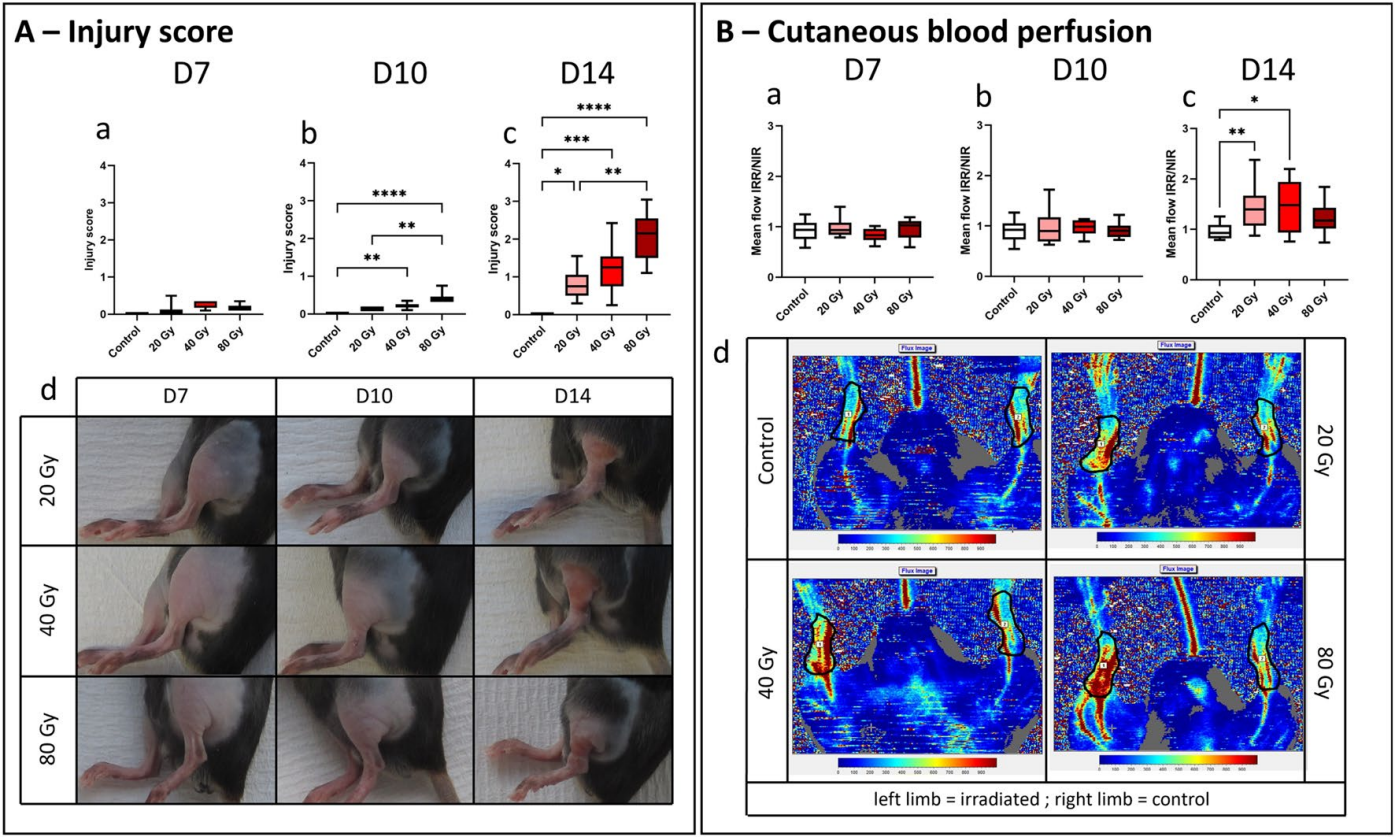
Injury follow-up. (**A**) Kinetics of the observational injury score. Box plots show the mean score from two experimenters for each group of doses at D7 (a) D10 (b) and D14 (c) post-irradiation. (d) Representative pictures of irradiated limb after 7, 10 and 14 days post-irradiation for each irradiated group (same mouse is represented for each group at each time point). (**B**) Monitoring of the cutaneous blood perfusion. Graphs show the mean ratio of the blood flow of the irradiated limb over the contralateral, non-irradiated one (IRR/NIR) at D7 (a), D10 (b) and D14 (c) post-irradiation. Representative Doppler scans of each dose group at day 14 post-irradiation are showed (d), with the area used for the analysis delimited in black. N = 15 for control, 20 and 80 Gy groups, N = 10 for 40 Gy group. Kruskal–Wallis test, *p < 0.05; **p < 0.01; ***p < 0.001; ****p < 0.0001.

The evolution of the severity of the injury was monitored by assigning an observational score (see Materials and Methods). At D7 post-irradiation, there was no clinical manifestation of LRI except for mild erythema on the limb for some irradiated mice. The irradiated groups could not be distinguished from each other and from non-irradiated mice based on clinical observation at this early timepoint (Fig. 2A, panels a & d). The injury evolved in a dose-dependent manner to higher severity from D10 to D14 post-irradiation for each dose group (Fig. 2A, panels b–d), with injury score significantly different between 20 and 80 Gy groups (1.48-fold, *p* = 0.0029). However, at D14, the injury score of the 40 Gy group was not statistically different from the other irradiated groups, despite a 0.55- and 0.6-fold difference compared to 20 Gy and 80 Gy groups, respectively (Fig. 2A, panel c).

At D7 and D10 post-irradiation, cutaneous blood perfusion analysis performed with laser Doppler showed no difference of the blood flow in irradiated limb compared to the contralateral one for each group (Fig. 2B, panels a & b). However, at D14 post-irradiation, a significant 50% increase of the cutaneous blood perfusion was observed in the irradiated limb compared to the non-irradiated one for the 40 Gy and 20 Gy groups, compared to the control group (*p* = 0.0036 and *p* = 0.0397, respectively; Fig. 2B, panel c & d). However, there was no significant difference between irradiation groups.

### Clinical and biological monitoring

**Figure 3:**
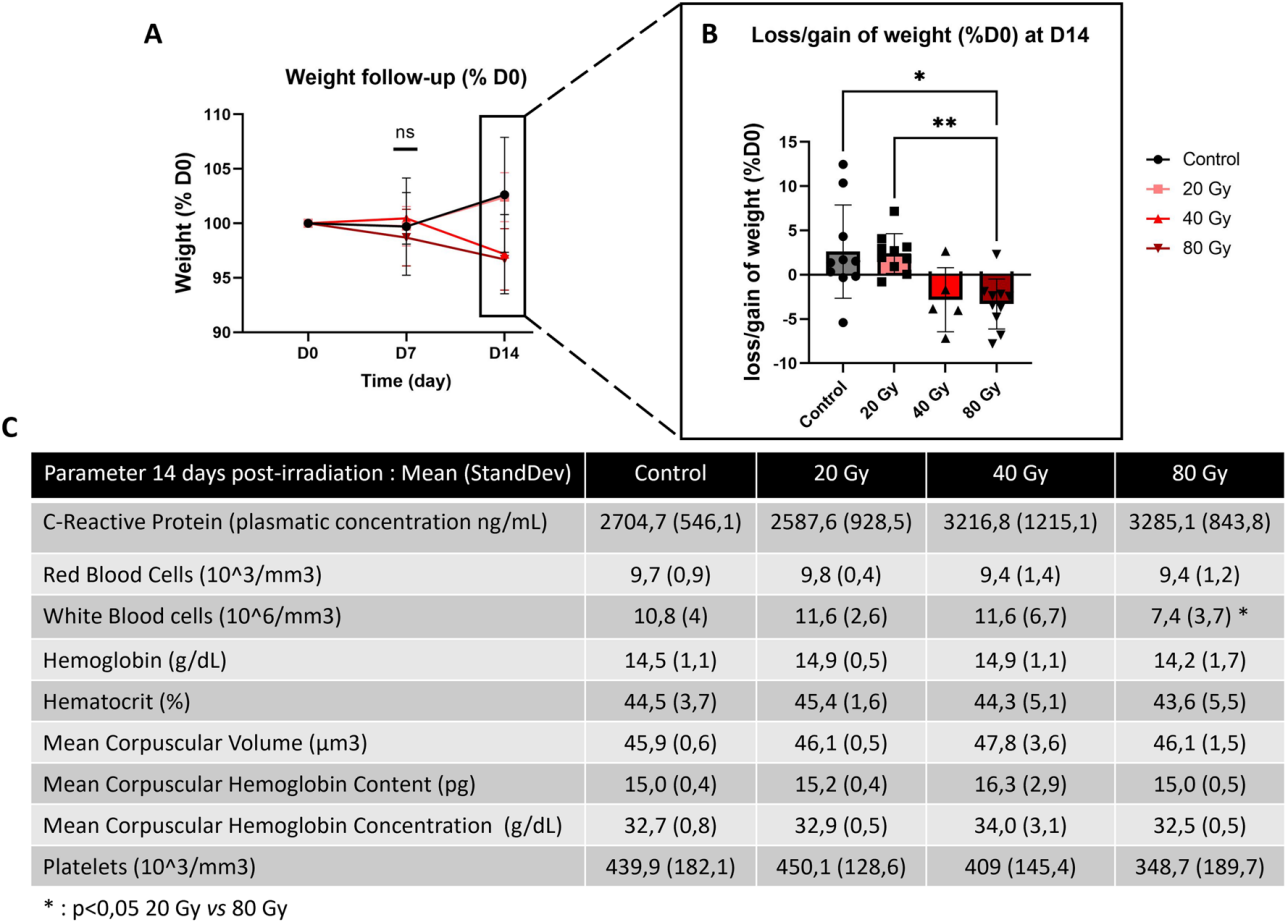
Clinical and biological analysis. (**A**) The weight monitoring was performed the day of irradiation (D0) and then 7 (D7) and 14 (D14) days after (Control, 20 Gy, 80 Gy: N = 10/group; 40 Gy: N = 5/group). (**B**) Weight gain or loss was calculated at day 14 post irradiation for each animal as a % of the weight change as compared to day 0. Mean +/− SEM for each group of doses; Control, 20 Gy, 80 Gy: N = 10/group; 40 Gy: N = 5/group). (**C**) Blood counts and plasmatic CRP levels measured at day 14 post-irradiation (Control, 20 Gy, 80 Gy: N = 15/group; 40 Gy: N = 10/group). Kruskal–Wallis test; *p < 0.05, **p < 0.001.

Animals exposed to higher doses displayed a trend toward a mild loss of weight at day 14 post-irradiation compared to D0 (Fig. 3A), with a mean loss of 2.8% for the 40 Gy group (not significant) and 3.3% for the 80 Gy group (*p* < 0.05 vs control group and *p* < 0.01 vs 20 Gy group) (Fig. 3B). The 20 Gy and control groups showed similar weight gain at D14. CBC showed no difference between groups except for a lower number of white blood cells (WBC) for the 80 Gy group compared to the 20 Gy group (*p* < 0.05) (Fig. 3C). Similarly, increased plasmatic concentrations of CRP were observed for the 40 Gy and 80 Gy groups compared to the 20 Gy and control groups, although not significant (Fig. 3C).

### Broad-spectrum miRNA analysis

**Figure 4:**
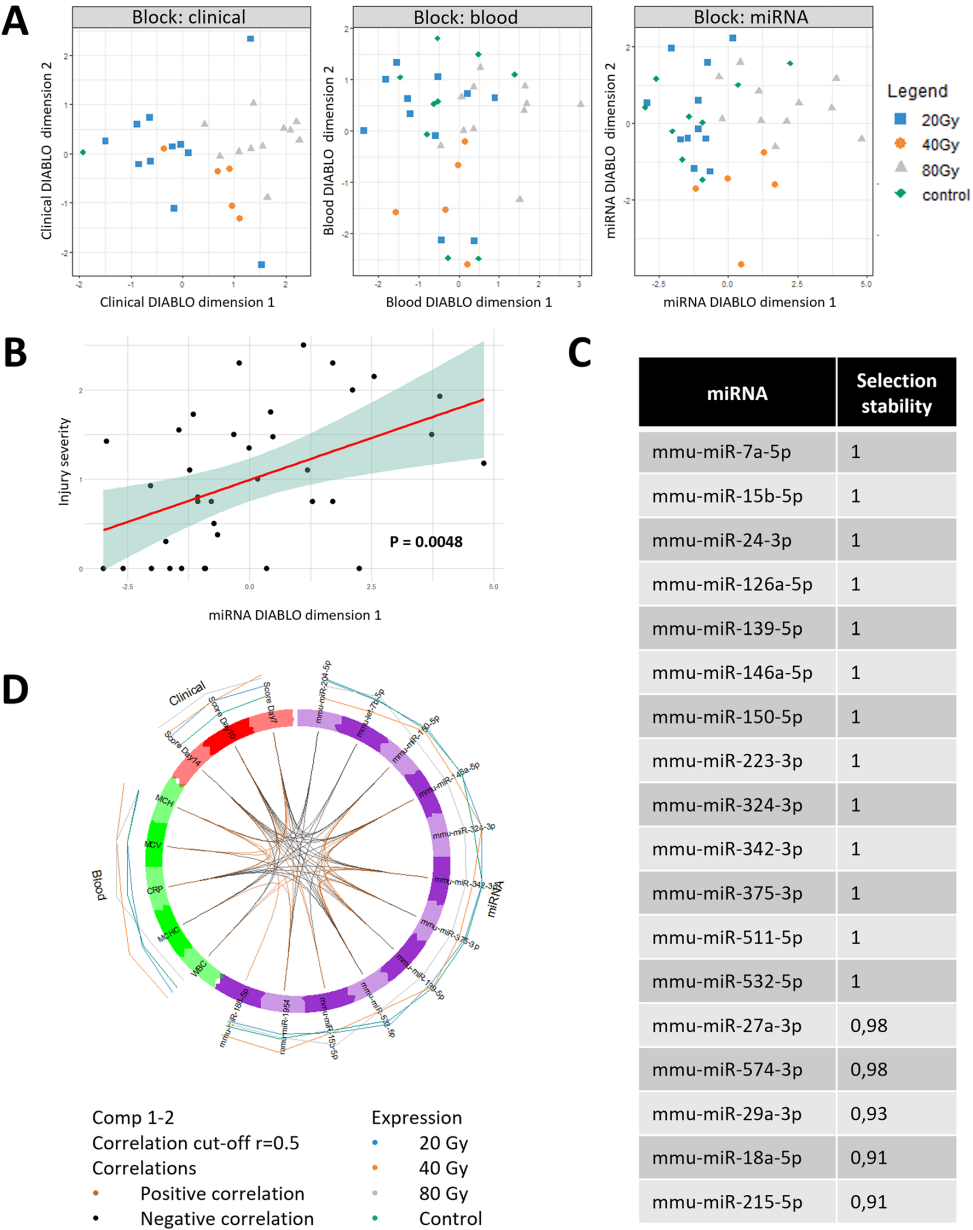
Broad-spectrum analysis. (**A**) Multi-block sPLS-DA scatter plots, with miRNA block = plasmatic expression of 18 miRNA among 750 miRNA analyzed; clinical block = injury score and cutaneous blood perfusion data at days 7, 10 and 14; blood block = results of CBC and plasmatic CRP level at D14. (**B**) Significant correlation between the first miRNA component of the DIABLO model with the injury severity at Day 14 after sPLS-DA, allowing to distinguish mice according to their injury severity and their group of dose (*p*-value = 0.0048, R^2^ = 0.19). (**C**) List of the 18 miRNAs selected (**D**) Circle plot providing a visualization of the multi-scale positive (orange) or negative (black) correlations between covariates of each block (estimated correlation greater than 0.5). These covariates are linked by a line inside the circle (colored according to the correlation sign). The lines outside the circle show the overall expression of the covariates across the dose groups. Control group N = 9, 20 Gy N = 11, 40 Gy N = 5, 80 Gy N = 11.

Broad-spectrum screening of 750 miRNAs analyzed by microfluidic array cards resulted in the robust identification of 320 miRNAs across all plasma samples. Univariate analyses on the association of the miRNAs with the dose groups and the injury score were conducted using permutation t-test and Spearman correlation, respectively. Several miRNAs showed significant association with dose groups or injury score, but none remained significant after Benjamini–Hochberg correction for multiple testing (see Supplementary Tables 2 and 3). Three blocks of covariates were defined for the multivariate analysis, including the miRNA block referring to plasmatic miRNA expression, the Clinical block to the injury score, and the Blood block to CBC and CRP levels (see Materials and Methods). The multivariate block PLS model confirmed the segregation of dose groups with the Clinical block (Fig. 4A), as previously seen with injury monitoring (Fig. 2A). Similar to results of biological monitoring (Fig. 3C), data from the Blood block did not permit to clearly separate the different groups. In contrast, following dimension reduction with DIABLO (see Materials and Methods), applying sPLS-DA on miRNA expression dataset led to a selection of 18 plasmatic miRNAs that was jointly able to distinguish animals according to their dose group (Fig. 4A) as well as exhibiting a significant correlation with the LRI injury scores (Fig. 4B, *p*-value = 0.0048). These 18 miRNAs (Fig. 4C) constitute the preliminary signature of LRI at D14 post-irradiation. The stability scores inform how often the same miRNAs are selected when the training set is perturbed via leave-one-out cross-validation. This information is important to evaluate the reproducibility of the miRNA signature. Thanks to the optimized inter-block correlations modeled by the DIABLO analysis, correlations between some of those miRNA expression levels and clinical parameters were identified, including CRP levels (for miR-146a-5p and miR-150-5p), WBC (miR-342-3p), and injury score at D14 (miR-139-5p and miR-532-5p) (Fig. 4D).

### Targeted analysis

**Figure 5:**
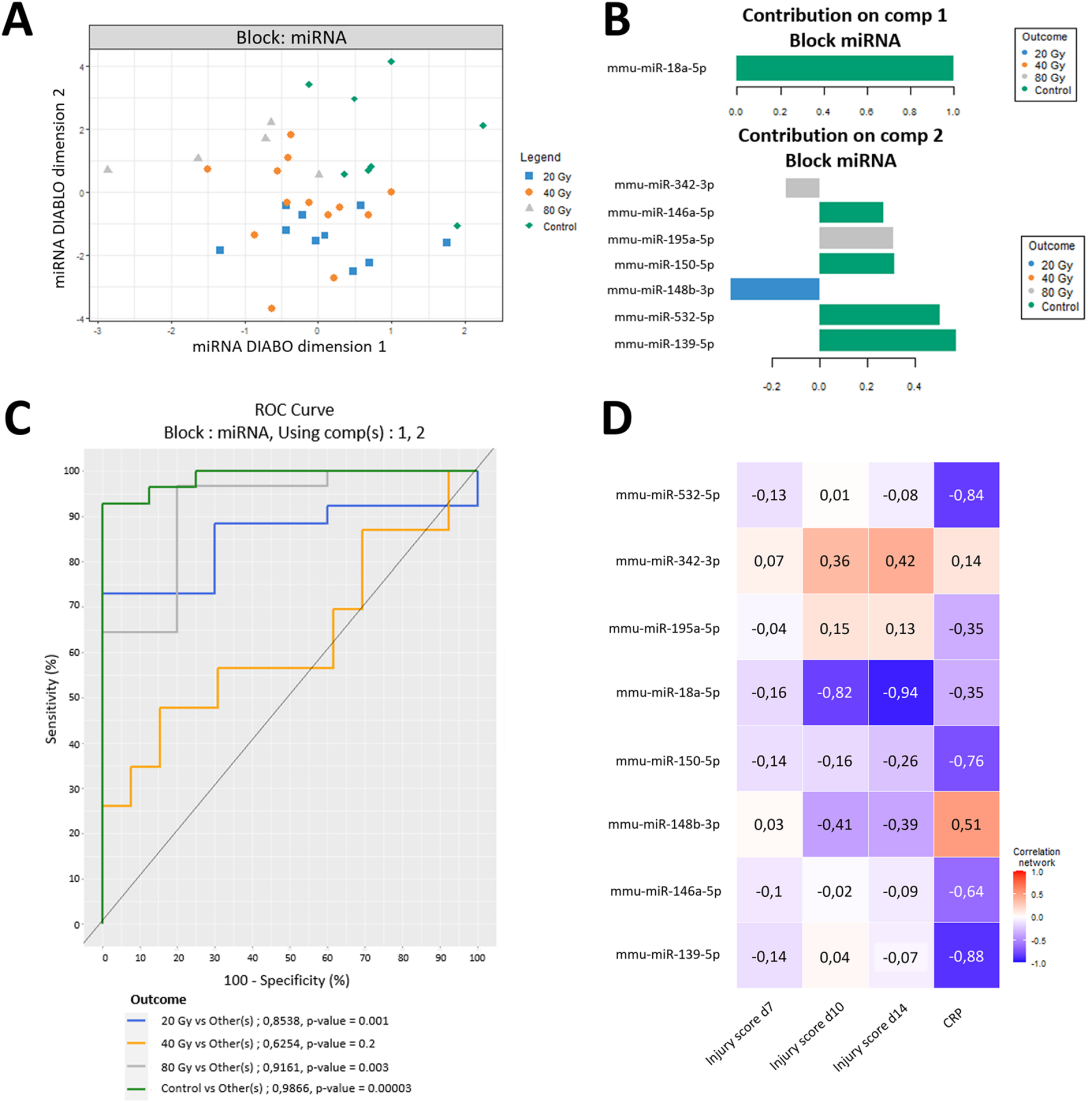
Validation analysis. (**A**) sPLS-DA using 8 validated miRNA is able to distinguish controls, 20 Gy and 80 Gy groups from each other, while the 40 Gy group appears to be intermediate. (**B**) Features included in sPLS analysis are plotted with their loading values. The loadings represent the contribution (coefficients in a linear combination) of the selected original miRNAs in the component. Colours indicate the dose group in which the median is maximum for each miRNA. (**C**) ROC curves representative of the 8-miRNA signature ability to distinguish each group of doses from the others, with the corresponding AUCs and *p*-values (outcome). (**D**) Heat map depicting correlations between miRNA plasma expression levels and the injury score (day 7, 10 and 14) and plasmatic CRP concentration (positive correlations in red and negatives in blue). Control group N = 8; 20 Gy N = 10; 40 Gy N = 13; 80 Gy N = 5.

The 18-miRNAs preliminary signature was further tested for validation in the plasma of irradiated and non-irradiated mice through a targeted analysis on an independent animal cohort (N = 15/group). The classification task of the DIABLO multi-block analysis showed the ability of an 8-miRNA subpanel to separate the control group from all irradiated groups (Fig. 5A). Furthermore, this restricted miRNA panel could well segregate the 20 Gy group and the 80 Gy group, but not the 40 Gy group from each other (Fig. 5A). Specifically, the loading plots showed that miR-18a-5p, miR-146a-5p, miR-150-5p, miR-532-5p and miR-139-5p mainly contributed to separate the control group from the others (Fig. 5B), while miR-342-3p and miR-195a-5p influenced the separation of the 80 Gy group. The 20 Gy group was essentially impacted by miR-148b-3p.

Receiver operating characteristic (ROC) curves with the 8-miRNA signature showed strong performance for the segregation of the control group, with an area under the curve (AUC) > 0.98, *p*-value = 0.00003), as well as the 80 Gy group (AUC > 0.91, *p*-value = 0.003) and the 20 Gy group (AUC > 0.85, *p*-value = 0.001; Fig. 5C). However, milder performances were achieved for the 40 Gy group (AUC = 0.6254, *p*-value = 0.2).

Similar to the broad-spectrum miRNA analysis, the obtained signature was also optimized to maximize the inter-block correlations. The reconstructed correlation matrix obtained by the DIABLO PLS multi-block components highlighted interesting multi-scale correlations. For example, circulating levels of miR-18a-5p were inversely correlated with the severity of the injury, while a positive correlation was found between injury score and miR-342-3p (Fig. 5D). In addition, some miRNAs, including miR-532-5p, miR-150-5p, miR-148b-3p, miR-146a-5p and miR-139-5p, exhibited individual correlation with CRP plasmatic levels.

Altogether, this study identified a mouse plasma signature composed of 8 miRNAs (Table 1) through a broad-spectrum analysis followed by a targeted study in a second animal cohort. This 8-miRNA signature can efficiently distinguish the irradiated animals from the non-irradiated ones and allows the segregation of animals with severe from mild radiation injuries.

**Table 1:** List of the 8 miRNAs composing the plasmatic diagnostic signature, their stability, fold-changes (* = adjusted *p*-value < 0.05, ** = adjusted *p*-value < 0.01) and putative functions in literature.

miRNA	Stability	Fold-change (irradiated vs control)	Putative function	References
miR-18a-5p	0.86	0.68**	Induction of keratinocytes apoptosis; disease markers of drug eruption in human	^17,61^
miR-139-5p	1	0.55**	Regulation of inflammatory skin wound in mice	^49^
miR-146a-5p	1	0.77*	Circulating radiation dosimeter for triage in NHPs	^20^
			Circulating biomarker of WBI in mice	^41^
			Regulation of inflammation in colon cancer cells	^62^
			Anti-fibrotic effect after acute muscle contusion in mice and muscle cells	^57^
miR-148b-3p	1	1.15	Negative regulator of inflammatory gene expression in IgA vasculitis adult patients	^63^
miR-150-5p	1	0.59**	Circulating biomarker of WBI in mice and patients	^43^
			Circulating radiation dosimeter for triage in NHPs	^20^
			Circulating biomarker of WBI in mice	^41^
			Marker of radiation response in cancer patients	^64^
			Reduction of inflammatory response and apoptosis of RAW264.7 macrophages after LPS stimulation	^65^
			Promotion of wound healing in HaCat keratinocytes	^55^
miR-195a-5p	1	0.98	Implicated in UVB-induced acute skin injury in mice	^16^
			Induces skeletal muscle wasting in C26 tumor bearing mice	^59^
miR-532-5p	1	0.66**	Regulation of inflammation in childhood asthma	^50^
			Regulation of inflammation in middle cerebral artery occlusion rat model	^66^
miR-342-3p	0.86	2.08*	Circulating radiation dosimeter for triage in NHPs	^20^
			Circulating indicator of WBI in mice	^44^

## Discussion

In situation of a radiological emergency, an external exposure to high doses of ionizing radiations may lead to LRI, which manifests after a latency phase as an erythema evolving from moist desquamation to deep ulceration and necrosis^4–7,29^. Early diagnosis and prognosis of victims of LRI is crucial for the medical management and the reduction of deleterious effects, by allowing victim surveillance, correct assignment to appropriate hospitals, and patient preparation for adapted therapeutic procedures. While several studies previously described omics biomarkers of WBI, including proteomic, transcriptomic, metabolomic and miRNA markers^30,31^, a limited number of studies addressed potential omics markers of LRI. Indeed, previous studies reported variations of tissue metabolites in a porcine model of cutaneous radiation injury^32^, proteome changes in mice serum after skin-only radiation exposure^33^, and tissue miRNA alterations in a mouse model of hindlimb radiation injury^34^. MiRNAs have also been studied as early biomarkers for diagnosis, prognosis, and treatment follow-up in different clinical settings, including the response to radiotherapy in cancer patients^35^, inflammation^36^, as well as cutaneous^37^ and muscular^38^ diseases. However, the potential use of circulating miRNAs in the context of LRI has not been documented. In order to identify early miRNA-based signatures predictive of the risk of developing LRI, we first conducted a proof-of-concept study using a preclinical model of hindlimb irradiation^39^ and we identified a plasmatic miRNA signature able to jointly discriminate the different dose groups and exhibit a significant association with injury severity.

The development of the injury in our LRI model was relevant to clinical symptoms, with a first asymptomatic phase during the first week after irradiation, followed by the appearance of the injury seven days post irradiation, and a dose-dependent aggravation up to day fourteen (end of experiment)^39^. The injury presented progressive and dose-dependent evolution of symptoms with the gradual emergence of erythema, oedema, dry then moist desquamation and ulceration, in accordance with the lesion kinetic described in human LRI cases^9^. Interestingly, at day 14 post-irradiation, the 40 Gy group was not statistically different from the other irradiated groups. This result could be explained by the heterogeneity of injury severity inside this dose group.

In contrast to WBI exposure, WBC counts were not relevant in our LRI model to characterize the injury severity or discriminate dose groups at D14 post-irradiation, despite lesions clearly established. Indeed, no correlation was found between the severity of LRI and these classical clinical measurements. This observation could be explained by the differences between our radiation exposure conditions and previous studies (global irradiation), where analysis was classically performed within the first 48 h ^20,21,40–44^. In addition, while laser Doppler monitoring of skin perfusion confirmed the radiation-induced increase in cutaneous blood flow, as previously described^39^, this parameter was unable to distinguish the radiation dose groups and did not show any significant correlation with injury severity at the timepoints analyzed in this study.

Considering that the aforementioned markers classically used in the clinical context of WBI assessment are not meaningful to reflect LRI manifestation, we hypothesized that molecular analyses would constitute a more sensitive approach in order to discriminate individuals with different degrees of LRI severity. Relying on this rationale, we designed a study to identify an LRI molecular diagnostic signature in blood through a two-step experimental design including a broad-spectrum profiling followed by a targeted step of a miRNA biomarkers subset to confirm the robustness of this signature. Broad-spectrum profiling of 750 miRNAs in plasma samples, combined with parsimonious multiblock analysis, provided the proof of concept of using miRNAs as plasmatic biomarkers of LRI, with the identification of an 18-miRNA panel able to distinguish animals according to their dose group and injury severity.

To our knowledge, this is the first-time systemic miRNA biomarkers were associated with localized irradiation. This miRNA signature showed a significant association with LRI (*p* = 0.0048) even with a moderate explained data variability (around 20%) which is not surprising in regard to the biological complexity of the radiation-induced cutaneous injuries. Interestingly, some of these 18 miRNAs (e.g. miR-150-5p, miR-342-3p, miR-146a-5p miR-532-5p) have already been identified as promising biomarkers of WBI in preclinical studies, with the ability to separate irradiated *vs* non irradiated individuals and segregate dose groups^20,41,44^. Moreover, the relevance of the miRNA signature is supported by multiscale correlations between macroscopic, clinical, and miRNA parameters, some of which had previously been described, such as CRP levels positively correlated with high grades of skin radiation injuries following radiotherapy in breast cancer patients^45^, or miR-146a-5p expression negatively correlated with CRP levels in patients with dermatomyositis^46^. Finally, the regulatory role of miR-150 on CRP levels was described in an acute myocardial infarction rat model^47^.

In order to strengthen our miRNA signature, we performed a targeted study of the 18-miRNA panel using an independent animal cohort. The multiscale analysis of this second dataset led to the validation of a final 8-miRNA panel that efficiently segregated animals according to their dose groups and injury severity. This validated signature, comprising miR-18a-5p, miR-139-5p, miR-146a-5p, miR-148b-3p, miR-150-5p, miR-195-5p, miR-532-5p, and miR-342-3p, showed strong performance to separate the control group, the 80 Gy group and the 20 Gy group from all other groups, although with lower performance to distinguish the 40 Gy group from the others. Interestingly, this inability at discriminating the 40 Gy group from the other dose groups was first observed when comparing the observational injury scores. Indeed, we noticed that the 40 Gy group exhibited a strong inter-individual variability, with animals displaying severe (respectively minor to moderate) injuries, similar to the 80 Gy group (respectively 20 Gy group; Fig. 2). Similarly, the multivariate analyses performed on the targeted study dataset could not allow a clear discrimination of this dose group. In this particular situation, the injury score variability leads to a reduction of the 40 Gy group classification task performance of the model in regard to its inter-block correlation optimization constraint. This observation demonstrates that our miRNA signature reflects tissue injury severity rather than irradiation dose and validates our strategy to include in our multiblock analysis all clinical parameters, including injury score, and not only dose groups. Besides, the poor discrimination ability for this intermediate dose (40 Gy) was previously reported in mouse serum proteomics analysis following skin radiation exposure^48^.

Interestingly, 4 miRNAs of our 8-miRNA signature have previously been identified as circulating biomarkers or potential biodosimeters of WBI in several preclinical studies. Indeed, Cui et al. demonstrated the proof of concept of the use of circulating miR-150-5p, miR-532-5p and miR-146a-5p as a predictive tool for radiation exposure in mice^41^. Similarly, miR-150-5p, miR-146a-5p and miR-342-3p were described as promising plasma candidate biomarkers in NHPs for radiological dosimetry^20^. Furthermore, miR-342-3p was reported as a potential early noninvasive indicator for irradiation in mice with a downregulation of its plasmatic expression 6–72 h after X-rays irradiation^44^. More recently, a study performed in mice after WBI and in patients receiving myeloablative radiotherapy reported on a molecular assay for the estimation of radiation dose based on the dose dependent depletion of circulating miR-150-5p^43^. Thus, the hypothesis of miRNA involvement in radiation response has been documented through several irradiation modalities both in preclinical models and cancer patients, and our study provides another line of evidence of the use of circulating miRNAs in radiation response after local radiation exposure.

Among the 8 miRNAs of the signature, 6 of them, namely miR-139-5p, miR-342-3p, miR-532-5p, miR-150-5p, miR-148b-3p and miR-146a-5p, have been described as regulators of inflammation in several studies. Indeed, an in vivo study demonstrated the role of miR-139-5p as a negative regulator of neutrophilic response in a preclinical model of bacteria infected wound healing^49^, while potential anti-inflammatory effect of miR-532-5p has been described in patients with asthma^50^ and in human macrophages in vitro^51^. Furthermore, miR-146a-5p and miR-150-5p have been described as circulating biomarkers in patients with different types of inflammatory diseases such as rheumatoid arthritis^52^ or sepsis^53^. As inflammation is part of LRI physiopathology^39,54^, the identification of miRNAs involved in inflammatory processes provides consistency to our signature.

Interestingly, some miRNAs of our panel have been associated with skin and muscle physiopathology which could confirm the relevance of our circulating signature. Indeed, miR-18a-5p was reported to induce apoptosis in keratinocytes, with a correlation between its serum expression level and the extent of skin erythema and erosion, suggesting its potential use as circulating marker in the context of severe cutaneous adverse reaction^17^. Similarly, miR-150-5p and miR-139-5p have been involved in skin wound healing^55,56^ and miR-195a-5p down-regulation after UVB radiation was related to acute skin injury^16^. In addition, some studies described miR-146a-5p implication in the mechanism of muscular fibrosis after acute contusion of skeletal muscle^57^ or cardiomyopathy^58^. Interestingly, miR-195a-5p has been shown to induce skeletal muscle wasting^59^, which has been associated with late stages of LRI^24^. This information suggests its important role in the development of severe LRI, which is supported by the strong contribution provided by miR-195a-5p to separate the 80 Gy group from the others in our study.

A limit of the present study is that we identified a circulating miRNA signature of LRI at the stage of manifest injury. Admittedly, such information would be more interesting when obtained during the latent, asymptomatic phase of the disease process. Benefiting from such an early molecular signature would provide invaluable predictive indication of the risk to experience LRI and its degree of severity. Further studies are ongoing to this aim, using our mouse preclinical model. Nonetheless, an accompanying miRNA diagnostic signature may prove useful when facing situations where the radiological nature of the injury is not suspected, or the scenario of the accident is not fully defined.

The specificity of our 8-miRNA signature for radiation-induced local injury, in regard to other stressors (chemical, thermal) has not been fully assessed, although most of the miRNAs present in this list have been associated with several physiological functions in different model organisms regarding response to ionizing radiation exposure, inflammation, apoptosis, or skin and muscle tissue pathophysiology. Further studies challenging this miRNA signature with other types of localized insult such as, for example, heat or chemical-induced musculocutaneous injuries, would help deciphering the specificity of this 8-miR panel for LRI.

Finally, translation towards human healthcare warrants further validation of predictive and diagnostic miRNA signatures by using other preclinical models of LRI, notably in large animal models such as minipig^32,60^.

## Conclusion

In contrast to previous studies focusing on a WBI molecular signature, we identified for the first time a plasmatic miRNA signature of LRI. The components of this circulating panel play a role in various functions involved in the LRI physiopathology such as inflammation, apoptosis, or wound healing, consistent with irradiation impact of key mechanisms in different tissue compartments such as skin or muscle. All this information reinforces the relevance of our circulating miRNA signature and highlight miRNAs as suitable molecular biomarkers to study the LRI. This proof-of-concept study constitutes a first step in the characterization of plasma miRNA signatures of LRI. Ongoing research aims at investigating earlier time points, *i.e.* before the appearance of first symptoms, in order to determine a circulating miRNA-based prognostic signature of LRI.

### Supplementary Information

Supplementary Information.

## Data Availability

The datasets generated and/or analyzed during the current study are available from the corresponding author on reasonable request.
