# Supplementary material for: microRNA blood signature for localized radiation injury
Source: Sci Rep. 2024 Feb 1;14:2681. doi: 10.1038/s41598-024-52258-2 (PMC10834964; doi:10.1038/s41598-024-52258-2)
Supplement: Supplementary file 1 — Supplementary Information. [file 41598_2024_52258_MOESM1_ESM.pdf]

microRNA blood signature for localized radiation injury

Lucie Ancel, Olivier Gabillot, Chloé Szurewsky, Romain Granger, Amandine Sache, Frédéric Voyer, Gaëtan Gruel, Stéphane Illiano, Marc Benderitter, Bernard Le Guen, Maâmar Souidi, Mohamed Amine Benadjaoud, Stéphane Flamant.

Supplementary information

Supplementary Figure 1: Injury scoring criteria.

Clinical criteria including erythema, lesion extent, oedema, desquamation, wound moisture and limb retraction were individually graded from 0 to 1 and summed to generate a global injury score.

Supplementary Table 1: Summary of individual injury scores.

Supplementary Table 2: Univariate analysis of the association of miRNAs with dose groups.

Supplementary Table 3: Univariate analysis of the association of miRNAs with D14 injury scores.

Supplementary figure 1. Injury scoring criteria

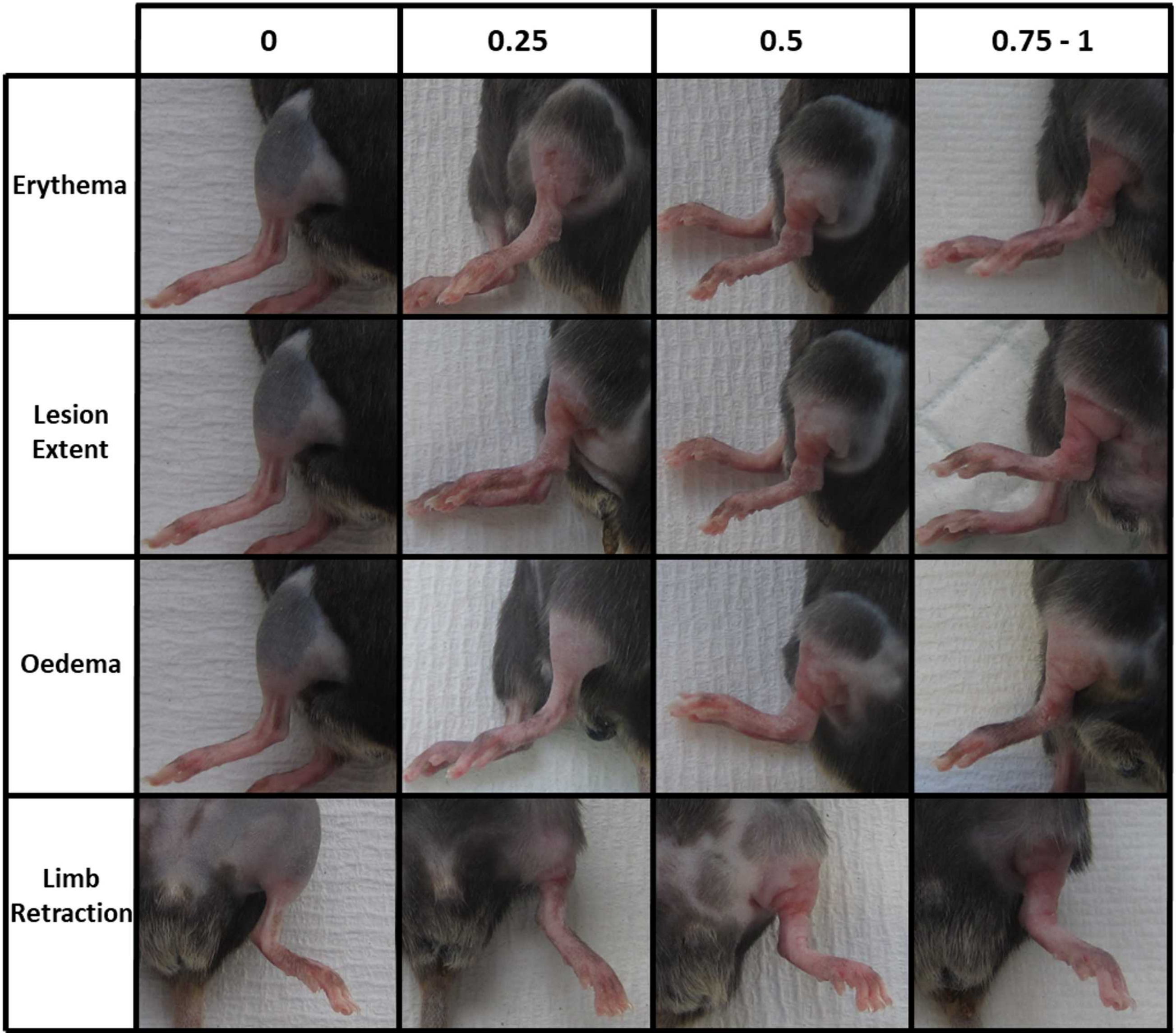

Supplementary Table 1: Summary of individual injury scores

Group	Animal	Erythema	Extent	Edema	Desquamation	Retraction	Total
Control	1	0	0	0	0	0	0
Control	2	0	0	0	0	0	0
Control	4	0	0	0	0	0	0
Control	5	0	0	0	0	0	0
Control	21	0	0	0	0	0	0
Control	22	0	0	0	0	0	0
Control	23	0	0	0	0	0	0
Control	24	0	0	0	0	0	0
Control	25	0	0	0	0	0	0
Control	41	0	0	0	0	0	0
Control	42	0	0	0	0	0	0
Control	43	0	0	0	0	0	0
Control	44	0	0	0	0	0	0
Control	45	0	0	0	0	0	0
20 Gy	6	0,25	0,25	0,25	0	0	0,75
20 Gy	7	0,25	0,25	0,25	0	0	0,75
20 Gy	8	0,25	0,25	0	0	0	0,5
20 Gy	9	0,25	0,25	0,25	0	0	0,75
20 Gy	10	0,25	0,25	0	0	0	0,5
20 Gy	26	0,25	0,25	0,25	0	0,25	1
20 Gy	27	0,175	0,25	0	0	0	0,425
20 Gy	28	0,25	0,25	0,175	0	0,25	0,925
20 Gy	29	0,25	0,25	0,375	0	0,25	1,125
20 Gy	30	0,1	0,175	0,1	0,175	0,1	0,65
20 Gy	46	0,375	0,25	0,25	0,1	0,25	1,225
20 Gy	47	0,375	0,25	0,25	0	0,175	1,05
20 Gy	48	0,625	0,375	0,25	0,1	0,25	1,6
20 Gy	49	0,25	0,25	0,175	0,1	0,375	1,15
20 Gy	50	0,625	0,375	0,25	0,1	0,25	1,6
40 Gy	12	0,25	0,25	0,5	0	0	1
40 Gy	13	0,5	0,25	0,25	0,1	0,25	1,35
40 Gy	14	0,25	0,25	0	0	0,25	0,75
40 Gy	15	0,25	0,25	0,25	0	0	0,75
40 Gy	51	1	0,25	0,625	0,1	0,5	2,475
40 Gy	52	0,75	0,25	0,1	0	0,375	1,475
40 Gy	53	0,5	0,375	0,375	0,1	0,25	1,6
40 Gy	54	0,5	0,25	0,175	0,1	0,175	1,2
40 Gy	55	0,625	0,25	0,375	0,1	0,375	1,725
80 Gy	16	0,25	0,25	0,25	0,1	0,25	1,1
80 Gy	17	0,25	0,25	0,5	0	0,5	1,5
80 Gy	18	0,5	0,25	0,5	0	0,5	1,75
80 Gy	19	0,5	0,25	0,5	0	0,25	1,5
80 Gy	20	0,5	0,5	0,5	0	0,5	2
80 Gy	36	0,75	0,25	0,375	0,175	0,75	2,3
80 Gy	37	1	0,25	0,625	0,2	1	3,075
80 Gy	38	0,875	0,25	0,625	0,2	0,625	2,575
80 Gy	39	0,625	0,25	0,75	0,2	0,875	2,7
80 Gy	40	1	0,25	0,5	0,25	0,75	2,75
80 Gy	56	1	0,25	0,5	0,1	0,5	2,35
80 Gy	57	0,875	0,25	0,375	0,1	0,375	1,975
80 Gy	58	1	0,25	0,5	0,1	0,35	2,2
80 Gy	59	0,375	0,25	0,375	0	0,175	1,175
80 Gy	60	1	0,25	0,625	0,1	0,625	2,6

Supplementary Table 2: Univariate analysis of the association of miRNAs with dose groups

Name	Dose	Fold-Change	Permutation Student p-value	Corrected Permutation Student p-value
mmu-miR-15b-5p	80Gy	2.6074	0.0017	0.35198
mmu-miR-875-5p	20Gy	0.2171	0.0022	0.35198
mmu-let-7i-5p	80Gy	1.7323	0.0027	0.35198
mmu-miR-130b-3p	20Gy	1.6544	0.003	0.35198
mmu-miR-146a-5p	80Gy	0.58727	0.0035	0.35198
mmu-miR-150-5p	80Gy	0.59012	0.0038	0.35198
mmu-miR-342-3p	80Gy	0.51426	0.0046	0.35198
mmu-miR-301b-3p	40Gy	1.617	0.0049	0.35198
mmu-miR-139-5p	80Gy	0.64889	0.0049	0.35198
mmu-miR-376b-5p	20Gy	0.25596	0.005	0.35198
mmu-miR-326-3p	80Gy	0.19215	0.0054	0.35198
mmu-miR-148b-3p	20Gy	1.5972	0.0074	0.42759
rno-miR-223-3p	80Gy	0.4335	0.0086	0.42759
mmu-miR-467b-3p	20Gy	0.46201	0.0092	0.42759
mmu-miR-1954	40Gy	2.7364	0.0098	0.42759
mmu-miR-872-3p	40Gy	0.39066	0.0099	0.42759
mmu-miR-375-3p	80Gy	0.36324	0.0105	0.42759
mmu-miR-24-3p	80Gy	0.6157	0.0111	0.42759
mmu-miR-345-5p	20Gy	1.7674	0.0114	0.42759
mmu-miR-532-5p	80Gy	0.69608	0.0123	0.42759
mmu-miR-223-3p	80Gy	0.62484	0.0134	0.42759
mmu-miR-20a-5p	20Gy	1.697	0.0134	0.42759
mmu-miR-16-5p	20Gy	1.6323	0.0142	0.42759
mmu-miR-106b-5p	20Gy	1.5668	0.0153	0.42759
mmu-miR-20b-5p	20Gy	1.5607	0.016	0.42759
mmu-let-7e-5p	80Gy	2.0036	0.0168	0.42759
mmu-miR-148b-3p	40Gy	1.5917	0.0178	0.42759
mmu-miR-181c-5p	40Gy	2.0983	0.0188	0.42759
mmu-miR-574-3p	80Gy	0.52715	0.019	0.42759
mmu-miR-324-3p	20Gy	1.543	0.0192	0.42759
mmu-miR-103-3p	40Gy	2.5205	0.021	0.42759
mmu-miR-7a-5p	80Gy	3.3076	0.0213	0.42759
mmu-miR-532-5p	40Gy	0.70866	0.0213	0.42759
mmu-miR-463-5p	80Gy	0.24296	0.0213	0.42759
mmu-miR-494-3p	80Gy	1.8123	0.0215	0.42759
mmu-miR-17-5p	40Gy	1.9097	0.0218	0.42759
mmu-let-7b-5p	40Gy	0.61633	0.0231	0.42759
mmu-miR-511-5p	80Gy	0.36153	0.0239	0.42759
mmu-miR-140-5p	20Gy	1.3813	0.0261	0.42759
mmu-miR-27a-3p	80Gy	0.66043	0.0263	0.42759
mmu-miR-463-3p	20Gy	0.37129	0.0268	0.42759
mmu-miR-24-3p	40Gy	0.66636	0.0284	0.42759
mmu-miR-455-3p	20Gy	0.37159	0.029	0.42759
mmu-miR-142-3p	80Gy	1.726	0.0291	0.42759
mmu-miR-130b-3p	80Gy	1.732	0.0296	0.42759
mmu-miR-872-3p	80Gy	0.44298	0.0298	0.42759
mmu-miR-875-5p	80Gy	0.2911	0.0299	0.42759
mmu-miR-204-5p	40Gy	0.25724	0.0305	0.42759
mmu-miR-345-5p	40Gy	1.6338	0.0306	0.42759
mmu-miR-193a-5p	40Gy	0.34005	0.0317	0.42759
mmu-miR-29a-3p	80Gy	0.77848	0.0322	0.42759
mmu-miR-7a-5p	40Gy	4.0572	0.0325	0.42759
mmu-miR-25-3p	20Gy	1.4375	0.0326	0.42759
mmu-miR-106a-5p	40Gy	1.4786	0.0326	0.42759
mmu-miR-18a-5p	20Gy	1.5714	0.0328	0.42759
mmu-miR-301a-3p	20Gy	1.3591	0.0335	0.42892
mmu-miR-126a-5p	80Gy	0.60659	0.0346	0.43523
mmu-miR-34b-3p	20Gy	0.43776	0.0355	0.43885
mmu-miR-19a-3p	20Gy	1.4103	0.0367	0.43892
mmu-miR-29a-5p	80Gy	0.37343	0.037	0.43892

Name	Dose	Fold-Change	Permutation Student p-value	Corrected Permutation Student p-value
rno-miR-532-5p	40Gy	0.72181	0.0374	0.43892
mmu-miR-434-3p	40Gy	0.61252	0.0382	0.43892
mmu-let-7g-5p	20Gy	1.3975	0.04	0.43892
mmu-miR-331-3p	20Gy	1.3252	0.0411	0.43892
mmu-miR-301b-3p	20Gy	1.4569	0.0412	0.43892
mmu-miR-24-2-5p	80Gy	0.56508	0.0413	0.43892
mmu-miR-451a	20Gy	1.6303	0.0416	0.43892
mmu-miR-338-5p	40Gy	0.47695	0.0424	0.43892
mmu-miR-30b-5p	20Gy	1.227	0.0424	0.43892
mmu-miR-3107-5p	20Gy	1.5243	0.044	0.43892
mmu-miR-455-3p	40Gy	0.52505	0.0441	0.43892
mmu-miR-215-5p	80Gy	0.50566	0.0443	0.43892
mmu-miR-29a-5p	40Gy	0.24083	0.0449	0.43892
mmu-miR-93-5p	20Gy	1.4271	0.0453	0.43892
mmu-miR-19b-3p	20Gy	1.3014	0.0461	0.44072
mmu-miR-106b-5p	40Gy	1.5332	0.0504	0.46404
mmu-miR-132-3p	40Gy	0.58125	0.0513	0.46404
mmu-miR-1961	20Gy	0.23002	0.0521	0.46404
mmu-miR-195a-5p	20Gy	1.452	0.0521	0.46404
mmu-miR-324-3p	80Gy	0.62201	0.0544	0.46404
mmu-miR-1971	20Gy	0.6058	0.0547	0.46404
mmu-miR-463-5p	20Gy	0.35926	0.055	0.46404
mmu-miR-142-3p	40Gy	1.6189	0.0556	0.46404
mmu-let-7d-5p	80Gy	1.7319	0.0557	0.46404
mmu-miR-15a-3p	80Gy	0.52603	0.0558	0.46404
mmu-miR-451a	40Gy	1.6367	0.0559	0.46404
rno-miR-345-3p	80Gy	0.65957	0.057	0.46404
mmu-miR-338-5p	20Gy	0.50306	0.0572	0.46404
mmu-miR-463-3p	80Gy	0.35916	0.0576	0.46404
mmu-miR-149-5p	20Gy	0.35135	0.0608	0.48164
mmu-miR-203-3p	80Gy	0.74825	0.0614	0.48164
mmu-miR-103-3p	80Gy	2.3177	0.0618	0.48164
mmu-miR-872-5p	80Gy	0.70278	0.0665	0.51269
mmu-miR-485-3p	20Gy	0.38143	0.0697	0.53165
mmu-miR-30e-3p	20Gy	0.44826	0.0706	0.53177
mmu-miR-30b-5p	40Gy	1.2431	0.0712	0.53177
mmu-miR-149-5p	80Gy	0.41923	0.0728	0.53666
mmu-miR-30b-5p	80Gy	1.203	0.0739	0.53666
mmu-miR-301a-3p	40Gy	1.3809	0.0741	0.53666
mmu-miR-30c-5p	20Gy	1.1367	0.0759	0.54165
mmu-miR-339-5p	80Gy	0.53197	0.0763	0.54165
mmu-miR-324-5p	80Gy	0.67996	0.0798	0.54193
mmu-miR-411-5p	80Gy	2.1115	0.0805	0.54193
mmu-miR-22-5p	80Gy	0.66597	0.0826	0.54193
mmu-miR-22-5p	20Gy	0.58073	0.0829	0.54193
mmu-miR-15a-5p	20Gy	1.7113	0.0836	0.54193
mmu-let-7i-5p	20Gy	1.3522	0.0837	0.54193
mmu-miR-455-5p	20Gy	0.49534	0.0847	0.54193
mmu-miR-200a-3p	40Gy	0.44869	0.0848	0.54193
mmu-miR-135b-5p	80Gy	0.46083	0.0862	0.54193
mmu-miR-204-5p	80Gy	0.59966	0.0863	0.54193
mmu-miR-29a-5p	20Gy	0.39582	0.0873	0.54193
mmu-miR-103-3p	20Gy	1.8475	0.0874	0.54193
mmu-miR-30e-3p	40Gy	0.44218	0.0876	0.54193
mmu-miR-451a	80Gy	1.4552	0.0889	0.54193
mmu-miR-29c-3p	40Gy	0.64271	0.0889	0.54193
mmu-miR-195a-5p	40Gy	1.422	0.0893	0.54193
mmu-miR-19a-3p	40Gy	1.4347	0.0895	0.54193
mmu-miR-205-5p	80Gy	0.55994	0.0901	0.54193
mmu-miR-214-5p	40Gy	0.42287	0.0907	0.54193
mmu-miR-494-3p	40Gy	1.7248	0.0926	0.5424
mmu-miR-193b-3p	20Gy	0.74954	0.0927	0.5424

Name	Dose	Fold-Change	Permutation Student p-value	Corrected Permutation Student p-value
mmu-miR-193a-5p	20Gy	0.55059	0.0941	0.5424
mmu-miR-22-3p	80Gy	0.59207	0.0947	0.5424
mmu-miR-1a-3p	80Gy	2.1	0.0953	0.5424
mmu-miR-151-3p	80Gy	0.65612	0.0954	0.5424
mmu-miR-92a-3p	20Gy	1.3064	0.0974	0.5424
mmu-miR-1971	80Gy	0.68235	0.0985	0.5424
mmu-miR-151-3p	20Gy	0.72998	0.0985	0.5424
rno-miR-632	20Gy	0.53993	0.0991	0.5424
mmu-miR-15a-5p	80Gy	2.1012	0.0991	0.5424
mmu-miR-331-3p	80Gy	1.4083	0.1026	0.55697
mmu-miR-146a-5p	40Gy	0.75472	0.1038	0.55697
rno-miR-223-3p	20Gy	0.661	0.1051	0.55697
mmu-miR-455-5p	40Gy	0.33972	0.1061	0.55697
mmu-miR-101a-3p	20Gy	1.2259	0.1062	0.55697
mmu-miR-23a-3p	80Gy	0.61541	0.1067	0.55697
mmu-miR-7b-5p	40Gy	1.6989	0.1072	0.55697
mmu-miR-193a-5p	80Gy	0.58114	0.1089	0.55875
mmu-miR-1191	80Gy	0.55083	0.1091	0.55875
mmu-miR-345-5p	80Gy	1.4464	0.1101	0.55906
mmu-miR-301a-3p	80Gy	1.2715	0.1112	0.55906
mmu-miR-30c-5p	80Gy	1.1285	0.1115	0.55906
mmu-miR-126a-3p	80Gy	0.73559	0.1178	0.58146
mmu-miR-331-3p	40Gy	1.3583	0.1184	0.58146
mmu-miR-212-3p	40Gy	0.31126	0.1184	0.58146
mmu-miR-1961	80Gy	0.26574	0.1203	0.58173
mmu-miR-199a-3p	40Gy	0.68149	0.1208	0.58173
mmu-miR-18a-5p	40Gy	1.5372	0.1211	0.58173
mmu-miR-155-5p	80Gy	0.68823	0.1217	0.58173
mmu-miR-712-5p	20Gy	0.43958	0.1246	0.59164
mmu-miR-574-3p	40Gy	0.65052	0.1276	0.59797
mmu-miR-376c-3p	40Gy	0.59126	0.1276	0.59797
mmu-miR-455-5p	80Gy	0.6052	0.1319	0.59848
mmu-miR-27b-3p	80Gy	0.72279	0.1319	0.59848
mmu-miR-673-3p	20Gy	0.61348	0.1322	0.59848
mmu-miR-494-3p	20Gy	1.5941	0.1323	0.59848
mmu-miR-484	20Gy	1.3211	0.1327	0.59848
mmu-miR-17-5p	20Gy	1.3549	0.1336	0.59848
mmu-miR-434-3p	20Gy	0.75043	0.1346	0.59848
mmu-miR-212-3p	20Gy	0.57454	0.136	0.59848
mmu-miR-26b-5p	20Gy	1.2169	0.1365	0.59848
mmu-miR-30e-3p	80Gy	0.54493	0.1367	0.59848
mmu-miR-1894-3p	80Gy	0.43961	0.1378	0.59848
rno-miR-223-3p	40Gy	0.63467	0.1385	0.59848
mmu-miR-350-3p	80Gy	0.61529	0.1393	0.59848
mmu-miR-410-3p	40Gy	2.6755	0.1411	0.59848
mmu-miR-491-5p	20Gy	1.3342	0.1414	0.59848
mmu-miR-342-3p	40Gy	0.67372	0.1417	0.59848
mmu-miR-24-2-5p	40Gy	0.58675	0.1419	0.59848
mmu-miR-126a-3p	40Gy	0.74064	0.1428	0.59876
mmu-miR-664-3p	40Gy	0.53417	0.1443	0.60153
mmu-miR-34c-3p	40Gy	3.3799	0.1462	0.60269
mmu-miR-99a-5p	20Gy	0.64674	0.1469	0.60269
mmu-miR-423-3p	20Gy	0.63962	0.1471	0.60269
mmu-miR-296-5p	20Gy	1.6332	0.1482	0.60375
rno-miR-7a-1-3p	80Gy	0.70244	0.1522	0.61654
mmu-miR-872-3p	20Gy	0.57868	0.1535	0.61831
mmu-miR-34a-5p	20Gy	0.76354	0.1583	0.63408
mmu-miR-192-5p	80Gy	0.66339	0.1593	0.63454
rno-miR-345-3p	40Gy	0.71493	0.1607	0.63659
mmu-miR-214-5p	80Gy	0.59841	0.1661	0.65436
mmu-miR-101a-3p	80Gy	1.1803	0.1677	0.65532
U6-snRNA	20Gy	1.8913	0.1687	0.65532

Name	Dose	Fold-Change	Permutation Student p-value	Corrected Permutation Student p-value
mmu-miR-192-5p	20Gy	0.73601	0.1691	0.65532
mmu-miR-145a-5p	80Gy	0.7819	0.17	0.65532
mmu-miR-3107-5p	80Gy	1.3383	0.171	0.65565
mmu-miR-744-5p	20Gy	1.1739	0.1766	0.66415
rno-miR-7a-1-3p	40Gy	0.55551	0.1767	0.66415
mmu-miR-24-3p	20Gy	0.79975	0.177	0.66415
mmu-miR-339-5p	40Gy	0.67956	0.1776	0.66415
mmu-miR-1839-5p	80Gy	0.58466	0.1787	0.66415
mmu-miR-29b-1-5p	20Gy	0.70031	0.1793	0.66415
mmu-miR-106b-5p	80Gy	1.2491	0.1797	0.66415
mmu-miR-410-3p	80Gy	1.7663	0.1838	0.66932
mmu-miR-22-5p	40Gy	0.65879	0.1838	0.66932
mmu-let-7c-5p	40Gy	0.81034	0.1839	0.66932
mmu-miR-376b-5p	80Gy	0.57866	0.1853	0.67093
rno-miR-148b-5p	80Gy	0.63564	0.1863	0.67093
mmu-miR-223-3p	20Gy	0.81032	0.188	0.67093
mmu-miR-191-5p	80Gy	0.8038	0.1885	0.67093
mmu-miR-425-3p	20Gy	0.26096	0.191	0.67093
mmu-miR-136-5p	40Gy	0.53348	0.1912	0.67093
mmu-miR-99b-5p	20Gy	0.69157	0.1913	0.67093
mmu-miR-146b-5p	40Gy	0.71986	0.193	0.67093
mmu-miR-1930-5p	20Gy	1.8992	0.1933	0.67093
mmu-miR-326-3p	20Gy	0.413	0.1937	0.67093
mmu-miR-205-5p	40Gy	0.66337	0.1966	0.67755
mmu-miR-338-5p	80Gy	0.68684	0.1975	0.67755
mmu-miR-2183	20Gy	0.58324	0.1995	0.68115
mmu-miR-7b-5p	20Gy	1.2858	0.2027	0.68622
mmu-miR-26b-3p	20Gy	0.57272	0.2029	0.68622
U6-snRNA	80Gy	1.9359	0.2045	0.68839
mmu-miR-26b-5p	40Gy	1.2039	0.2079	0.69656
mmu-miR-10a-5p	80Gy	0.48033	0.2096	0.69899
mmu-miR-340-5p	80Gy	0.82049	0.2122	0.70354
mmu-miR-335-5p	20Gy	0.7542	0.2145	0.70354
mmu-miR-491-5p	40Gy	1.4651	0.2151	0.70354
mmu-miR-301b-3p	80Gy	1.2658	0.2152	0.70354
mmu-miR-664-3p	80Gy	0.75513	0.216	0.70354
mmu-miR-127-3p	40Gy	0.67313	0.2176	0.70354
mmu-miR-24-2-5p	20Gy	0.67744	0.2185	0.70354
mmu-miR-485-3p	40Gy	0.5584	0.2198	0.70354
mmu-miR-296-5p	40Gy	2.2725	0.2225	0.70354
mmu-let-7a-1-3p	80Gy	0.66451	0.2228	0.70354
mmu-miR-423-5p	20Gy	1.3067	0.2231	0.70354
mmu-miR-652-3p	20Gy	1.23	0.2233	0.70354
rno-miR-196c-5p	20Gy	1.6973	0.2244	0.70354
mmu-miR-194-5p	80Gy	1.4782	0.2247	0.70354
mmu-miR-224-5p	40Gy	0.68532	0.226	0.70453
mmu-miR-126a-3p	20Gy	0.79366	0.2273	0.70552
mmu-miR-181c-5p	20Gy	1.3345	0.2285	0.70618
mmu-miR-29b-3p	80Gy	1.7626	0.2318	0.71331
mmu-miR-185-5p	40Gy	1.3423	0.2329	0.71363
rno-miR-224-5p	20Gy	0.64951	0.2371	0.71759
mmu-miR-10a-5p	40Gy	0.45895	0.2388	0.71759
mmu-miR-28a-5p	20Gy	0.75351	0.2389	0.71759
mmu-miR-15b-3p	80Gy	0.58621	0.2412	0.71759
mmu-miR-1969	40Gy	2.2861	0.2416	0.71759
mmu-miR-1894-3p	20Gy	0.48491	0.2421	0.71759
mmu-miR-214-5p	20Gy	0.65353	0.2426	0.71759
mmu-miR-210-3p	80Gy	0.81946	0.2429	0.71759
mmu-miR-185-5p	20Gy	1.2489	0.2432	0.71759
mmu-miR-139-5p	20Gy	0.8782	0.2458	0.72229
rno-miR-632	80Gy	0.58754	0.2477	0.7249
mmu-miR-467b-3p	80Gy	0.6526	0.25	0.72866

Name	Dose	Fold-Change	Permutation Student p-value	Corrected Permutation Student p-value
mmu-miR-100-5p	80Gy	1.8985	0.2528	0.73162
mmu-miR-19a-3p	80Gy	1.1859	0.2533	0.73162
mmu-miR-15a-5p	40Gy	1.4655	0.2551	0.73162
mmu-miR-210-3p	40Gy	0.58868	0.2584	0.73162
mmu-miR-1191	20Gy	0.6653	0.2594	0.73162
mmu-miR-673-3p	40Gy	1.7828	0.2611	0.73162
mmu-miR-34a-5p	40Gy	0.80436	0.2618	0.73162
mmu-miR-375-3p	40Gy	0.59741	0.2622	0.73162
mmu-miR-324-3p	40Gy	1.5088	0.2624	0.73162
mmu-miR-574-3p	20Gy	0.77021	0.2625	0.73162
mmu-miR-10a-5p	20Gy	0.48464	0.2635	0.73162
mmu-miR-16-5p	80Gy	1.2847	0.2642	0.73162
mmu-miR-9-5p	80Gy	1.7792	0.2652	0.73162
mmu-miR-30e-5p	20Gy	1.1262	0.2653	0.73162
mmu-miR-22-3p	40Gy	0.53126	0.2696	0.73648
mmu-miR-29b-1-5p	80Gy	0.67574	0.2717	0.73648
mmu-miR-146b-5p	80Gy	0.80148	0.2736	0.73648
mmu-miR-181a-1-3p	80Gy	1.6578	0.2756	0.73648
mmu-miR-322-5p	40Gy	1.4762	0.2757	0.73648
mmu-miR-25-3p	80Gy	1.1825	0.277	0.73648
mmu-miR-378a-5p	80Gy	1.4098	0.2772	0.73648
mmu-miR-214-3p	20Gy	0.75189	0.2778	0.73648
mmu-miR-188-5p	20Gy	0.70338	0.2779	0.73648
mmu-miR-324-3p-1	40Gy	2.2338	0.2802	0.73648
mmu-miR-99a-5p	40Gy	1.5456	0.2805	0.73648
mmu-let-7a-1-3p	20Gy	0.65541	0.2812	0.73648
mmu-miR-29a-3p	20Gy	0.8892	0.2818	0.73648
rno-miR-345-3p	20Gy	0.70611	0.282	0.73648
mmu-miR-361-5p	20Gy	0.6856	0.283	0.73648
rno-miR-322-3p	80Gy	0.42013	0.2835	0.73648
mmu-miR-222-3p	20Gy	1.203	0.2846	0.73667
mmu-miR-696	80Gy	0.6234	0.288	0.74064
mmu-miR-144-3p	80Gy	1.3278	0.2882	0.74064
mmu-miR-2183	40Gy	1.7551	0.2906	0.74414
mmu-miR-485-3p	80Gy	0.65237	0.2928	0.74618
rno-miR-214-3p	80Gy	0.68756	0.2962	0.74618
mmu-miR-350-3p	40Gy	0.63303	0.2962	0.74618
mmu-miR-30a-3p	40Gy	0.65771	0.2964	0.74618
mmu-miR-532-5p	20Gy	1.1064	0.2966	0.74618
mmu-miR-374b-5p	80Gy	0.53881	0.3007	0.74647
mmu-miR-149-5p	40Gy	0.46966	0.3009	0.74647
mmu-miR-206-3p	80Gy	0.61291	0.3013	0.74647
mmu-miR-200c-3p	80Gy	1.3868	0.3021	0.74647
mmu-miR-140-3p	20Gy	1.5747	0.3036	0.74647
mmu-miR-101a-3p	40Gy	1.2158	0.3037	0.74647
mmu-miR-340-5p	20Gy	1.1628	0.304	0.74647
mmu-miR-802-5p	40Gy	1.6601	0.3079	0.75129
mmu-miR-467b-3p	40Gy	1.5228	0.3087	0.75129
mmu-miR-133b-3p	80Gy	1.523	0.3098	0.75129
mmu-miR-17-5p	80Gy	1.1987	0.311	0.75129
mmu-miR-379-5p	20Gy	0.58517	0.3114	0.75129
mmu-miR-335-5p	40Gy	1.3744	0.3132	0.75129
mmu-miR-126a-5p	40Gy	0.77443	0.3133	0.75129
mmu-miR-328-3p	80Gy	0.82014	0.3172	0.75524
mmu-miR-138-5p	40Gy	0.7559	0.3174	0.75524
mmu-miR-99a-5p	80Gy	1.4601	0.3199	0.75524
mmu-miR-130a-3p	80Gy	0.80115	0.3226	0.75524
mmu-miR-365-3p	20Gy	0.86807	0.3232	0.75524
mmu-miR-379-5p	40Gy	0.57911	0.3246	0.75524
mmu-miR-652-3p	80Gy	0.84188	0.3248	0.75524
mmu-miR-18a-3p	80Gy	0.73147	0.3257	0.75524
mmu-let-7d-5p	40Gy	1.3035	0.3264	0.75524

Name	Dose	Fold-Change	Permutation Student p-value	Corrected Permutation Student p-value
mmu-miR-1839-3p	80Gy	0.77243	0.3267	0.75524
mmu-miR-374b-5p	20Gy	0.5601	0.3271	0.75524
rno-miR-200b-3p	80Gy	0.65258	0.3296	0.75524
mmu-miR-324-5p	40Gy	1.4428	0.3311	0.75524
mmu-miR-130a-3p	40Gy	0.80933	0.3311	0.75524
mmu-let-7i-5p	40Gy	1.271	0.3313	0.75524
mmu-miR-30d-5p	40Gy	0.90149	0.3318	0.75524
mmu-miR-221-3p	20Gy	1.4089	0.3337	0.75688
mmu-miR-142-5p	20Gy	1.2698	0.3372	0.75688
mmu-miR-186-5p	80Gy	1.2461	0.3375	0.75688
mmu-miR-23b-3p	80Gy	0.66315	0.3378	0.75688
mmu-miR-1961	40Gy	0.39697	0.3378	0.75688
mmu-miR-29c-3p	20Gy	0.8087	0.341	0.75928
mmu-miR-135a-5p	40Gy	0.6949	0.3417	0.75928
mmu-miR-491-5p	80Gy	1.1801	0.3426	0.75928
mmu-miR-31-3p	80Gy	0.57655	0.3437	0.75928
mmu-miR-93-3p	80Gy	0.76695	0.3447	0.75928
mmu-miR-20a-5p	80Gy	1.1913	0.3474	0.75928
rno-miR-381-3p	40Gy	0.41503	0.3481	0.75928
mmu-miR-802-5p	20Gy	0.73667	0.3481	0.75928
mmu-miR-152-3p	80Gy	0.84705	0.3484	0.75928
mmu-miR-100-5p	40Gy	0.75339	0.3509	0.75985
mmu-miR-1191	40Gy	0.68312	0.3515	0.75985
mmu-miR-221-3p	40Gy	0.68866	0.3528	0.75985
mmu-miR-155-5p	20Gy	0.82166	0.3529	0.75985
mmu-miR-188-5p	40Gy	2.0774	0.3559	0.76401
rno-miR-200b-3p	20Gy	0.64529	0.3583	0.76687
mmu-miR-1894-3p	40Gy	0.50056	0.3608	0.76992
rno-miR-148b-5p	20Gy	0.72654	0.362	0.77019
mmu-miR-212-3p	80Gy	0.70859	0.3642	0.7703
mmu-miR-133a-3p	80Gy	1.4089	0.3642	0.7703
mmu-miR-126a-5p	20Gy	0.82567	0.3668	0.77352
mmu-miR-16-5p	40Gy	1.2545	0.3694	0.7751
mmu-miR-29b-1-5p	40Gy	1.4332	0.3698	0.7751
mmu-miR-25-3p	40Gy	1.2372	0.371	0.7751
mmu-miR-125a-5p	20Gy	0.84047	0.3744	0.7751
mmu-miR-218-5p	20Gy	0.85085	0.3749	0.7751
mmu-miR-429-3p	20Gy	0.6939	0.3751	0.7751
mmu-miR-23a-3p	40Gy	0.76633	0.3752	0.7751
mmu-miR-532-3p	40Gy	0.7764	0.3762	0.7751
mmu-miR-145a-5p	20Gy	0.82825	0.3787	0.77802
rno-miR-146b-5p	80Gy	0.73092	0.3801	0.77866
mmu-miR-206-3p	20Gy	0.66495	0.3829	0.78085
mmu-let-7c-5p	80Gy	1.1502	0.3844	0.78085
mmu-miR-365-3p	40Gy	0.84489	0.3848	0.78085
rno-miR-200b-3p	40Gy	0.53036	0.3859	0.78085
mmu-miR-361-5p	80Gy	0.60754	0.3869	0.78085
mmu-miR-1897-5p	40Gy	1.5264	0.3877	0.78085
mmu-miR-26a-5p	20Gy	0.82252	0.3926	0.7885
U6-snRNA	40Gy	1.4013	0.3942	0.7895
mmu-miR-93-5p	80Gy	1.1336	0.3959	0.7907
rno-miR-196c-5p	40Gy	0.62914	0.3979	0.79248
mmu-miR-652-3p	40Gy	0.73976	0.402	0.79487
mmu-miR-23a-3p	20Gy	0.82822	0.4023	0.79487
mmu-miR-1839-5p	20Gy	0.77965	0.4061	0.79487
rno-miR-214-3p	40Gy	0.71668	0.4064	0.79487
mmu-miR-199b-5p	20Gy	0.65192	0.4071	0.79487
mmu-miR-376b-5p	40Gy	0.6806	0.4078	0.79487
mmu-miR-1954	20Gy	0.72182	0.4083	0.79487
mmu-miR-34c-3p	80Gy	1.5057	0.4084	0.79487
mmu-miR-511-5p	40Gy	0.71247	0.4101	0.79487
mmu-miR-18a-3p	40Gy	1.3199	0.4109	0.79487

Name	Dose	Fold-Change	Permutation Student p-value	Corrected Permutation Student p-value
mmu-miR-335-3p	20Gy	0.79624	0.412	0.79487
mmu-miR-674-3p	20Gy	0.75775	0.4124	0.79487
mmu-miR-93-5p	40Gy	1.2468	0.4143	0.79616
mmu-miR-210-3p	20Gy	1.1277	0.4156	0.79616
mmu-miR-322-5p	20Gy	0.81025	0.4164	0.79616
mmu-miR-34b-3p	40Gy	0.59376	0.4202	0.8003
mmu-miR-141-3p	80Gy	1.2207	0.4208	0.8003
mmu-miR-877-3p	80Gy	0.69625	0.4223	0.80103
mmu-miR-130a-3p	20Gy	0.89877	0.4252	0.8044
mmu-miR-21a-5p	40Gy	1.1435	0.4277	0.807
mmu-miR-425-3p	80Gy	0.36415	0.4302	0.80959
mmu-miR-138-1-3p	80Gy	1.8367	0.435	0.80983
mmu-miR-30e-5p	40Gy	1.1138	0.4353	0.80983
mmu-miR-429-3p	40Gy	0.58167	0.4358	0.80983
mmu-miR-7a-1-3p	80Gy	0.81889	0.4387	0.80983
mmu-miR-27b-3p	20Gy	0.84012	0.4388	0.80983
mmu-miR-421-3p	80Gy	0.81026	0.4404	0.80983
mmu-miR-374b-5p	40Gy	0.47592	0.4407	0.80983
mmu-miR-455-3p	80Gy	0.69454	0.4435	0.80983
mmu-miR-190b-5p-bis	40Gy	0.77455	0.4456	0.80983
mmu-miR-30a-3p	80Gy	0.78407	0.4458	0.80983
mmu-miR-532-3p	80Gy	0.89248	0.4482	0.80983
mmu-miR-664-3p	20Gy	0.80657	0.4491	0.80983
mmu-miR-20b-5p	80Gy	1.1444	0.4491	0.80983
mmu-miR-142-3p	20Gy	1.2092	0.4499	0.80983
mmu-miR-200b-3p	80Gy	1.3982	0.451	0.80983
mmu-miR-138-1-3p	20Gy	1.8044	0.4516	0.80983
rno-miR-1-3p	20Gy	0.75672	0.4523	0.80983
mmu-miR-495-3p	40Gy	1.3555	0.4534	0.80983
mmu-miR-192-5p	40Gy	0.74916	0.4537	0.80983
mmu-miR-32-5p	20Gy	1.1091	0.4548	0.80983
mmu-miR-16-1-3p	80Gy	0.56278	0.4549	0.80983
mmu-miR-193b-3p	40Gy	0.86235	0.4588	0.80983
rno-miR-381-3p	80Gy	0.73881	0.459	0.80983
mmu-miR-20a-5p	40Gy	1.2613	0.4591	0.80983
mmu-miR-30d-5p	80Gy	1.083	0.4606	0.80983
mmu-miR-29a-3p	40Gy	0.8859	0.4626	0.80983
mmu-miR-148a-3p	40Gy	1.2458	0.464	0.80983
mmu-miR-676-3p	40Gy	0.83578	0.4649	0.80983
mmu-miR-152-3p	20Gy	0.87931	0.4649	0.80983
mmu-miR-135b-5p	40Gy	1.746	0.4665	0.80983
mmu-miR-142-5p	80Gy	1.2028	0.4668	0.80983
mmu-miR-1839-5p	40Gy	0.63121	0.4669	0.80983
mmu-miR-31-3p	40Gy	0.60429	0.4676	0.80983
mmu-miR-27a-3p	20Gy	0.88167	0.4712	0.8141
rno-miR-1-3p	80Gy	1.2566	0.4728	0.8149
mmu-miR-322-5p	80Gy	0.82288	0.4754	0.81741
mmu-miR-186-5p	40Gy	1.1984	0.482	0.82678
mmu-miR-423-3p	80Gy	0.80129	0.4878	0.82742
mmu-miR-15b-5p	20Gy	1.2054	0.4878	0.82742
mmu-miR-106b-3p	80Gy	0.73118	0.4878	0.82742
mmu-miR-181a-5p	20Gy	0.82842	0.4886	0.82742
mmu-miR-744-5p	80Gy	0.92576	0.4889	0.82742
mmu-miR-186-5p	20Gy	1.1747	0.4893	0.82742
mmu-miR-21a-5p	80Gy	0.89265	0.4963	0.83729
mmu-miR-9-5p	20Gy	1.303	0.4977	0.83768
mmu-miR-106a-5p	20Gy	1.1165	0.501	0.83921
mmu-miR-30c-5p	40Gy	1.0841	0.5023	0.83921
mmu-miR-423-3p	40Gy	1.2288	0.5034	0.83921
mmu-miR-9-3p	80Gy	1.3883	0.505	0.83921
mmu-miR-744-5p	40Gy	1.1353	0.506	0.83921
rno-miR-224-5p	40Gy	0.76928	0.5077	0.83921

Name	Dose	Fold-Change	Permutation Student p-value	Corrected Permutation Student p-value
mmu-miR-200a-3p	80Gy	0.83152	0.5078	0.83921
mmu-miR-224-5p	80Gy	0.81728	0.5109	0.83921
rno-miR-214-3p	20Gy	0.78564	0.5117	0.83921
mmu-miR-138-1-3p	40Gy	1.7922	0.5139	0.83921
mmu-miR-181c-5p	80Gy	0.85945	0.5143	0.83921
mmu-miR-30a-5p	80Gy	0.94235	0.5144	0.83921
mmu-miR-106b-3p	20Gy	1.3085	0.5159	0.83921
mmu-miR-193b-3p	80Gy	0.89494	0.5171	0.83921
mmu-miR-326-3p	40Gy	2.1618	0.5177	0.83921
mmu-miR-500-3p	80Gy	1.3367	0.5241	0.83921
mmu-miR-7a-1-3p	40Gy	1.1649	0.5254	0.83921
mmu-miR-106a-5p	80Gy	1.1383	0.5268	0.83921
mmu-miR-32-5p	40Gy	0.8745	0.527	0.83921
mmu-miR-22-3p	20Gy	0.7567	0.5271	0.83921
mmu-let-7b-5p	80Gy	1.1333	0.5275	0.83921
mmu-miR-16-1-3p	20Gy	0.6202	0.5288	0.83921
mmu-miR-136-5p	20Gy	0.78316	0.529	0.83921
mmu-miR-365-3p	80Gy	0.91452	0.5292	0.83921
mmu-miR-20b-5p	40Gy	0.68888	0.5315	0.83921
mmu-miR-218-5p	40Gy	0.88155	0.5316	0.83921
mmu-miR-425-3p	40Gy	0.39744	0.5331	0.83921
mmu-miR-1896	20Gy	0.74664	0.5334	0.83921
mmu-miR-99b-5p	40Gy	1.2552	0.5376	0.83921
mmu-miR-144-3p	20Gy	1.2256	0.5421	0.83921
mmu-miR-138-5p	20Gy	0.87064	0.5425	0.83921
mmu-miR-184-3p	20Gy	0.60037	0.5428	0.83921
mmu-miR-218-5p	80Gy	1.1408	0.5429	0.83921
mmu-miR-696	20Gy	0.80765	0.5449	0.83921
mmu-miR-188-5p	80Gy	0.81535	0.5453	0.83921
mmu-miR-125a-5p	40Gy	0.86841	0.5457	0.83921
mmu-miR-7b-5p	80Gy	1.129	0.5478	0.83921
mmu-let-7a-1-3p	40Gy	0.80254	0.5482	0.83921
rno-miR-196c-5p	80Gy	1.3614	0.5491	0.83921
mmu-miR-872-5p	40Gy	0.84369	0.5496	0.83921
mmu-miR-144-3p	40Gy	1.1963	0.5499	0.83921
mmu-miR-362-3p	80Gy	0.85848	0.5511	0.83921
mmu-miR-195a-5p	80Gy	1.1016	0.5524	0.83921
mmu-miR-29b-3p	40Gy	1.3317	0.5532	0.83921
mmu-miR-99b-5p	80Gy	1.2088	0.5548	0.83921
mmu-miR-203-3p	40Gy	0.84696	0.5548	0.83921
mmu-miR-141-3p	20Gy	0.84413	0.5558	0.83921
mmu-miR-190b-5p-bis	80Gy	0.84146	0.5564	0.83921
mmu-miR-181a-5p	40Gy	1.2509	0.557	0.83921
mmu-miR-138-5p	80Gy	0.83657	0.5572	0.83921
mmu-miR-122-5p	20Gy	0.82665	0.5583	0.83921
mmu-let-7g-5p	80Gy	1.0875	0.5616	0.83923
mmu-miR-190b-5p-bis	20Gy	0.85455	0.5617	0.83923
mmu-miR-152-3p	40Gy	0.88622	0.5624	0.83923
rno-miR-532-5p	80Gy	0.86562	0.563	0.83923
mmu-miR-1896	40Gy	1.4601	0.565	0.84047
mmu-miR-31-3p	20Gy	0.7538	0.5699	0.846
mmu-miR-23b-3p	40Gy	0.81583	0.5768	0.8494
mmu-miR-378a-5p	20Gy	0.83331	0.5773	0.8494
mmu-miR-106b-3p	40Gy	0.72086	0.5775	0.8494
mmu-miR-335-5p	80Gy	0.88974	0.5782	0.8494
mmu-miR-23b-3p	20Gy	0.83365	0.5784	0.8494
mmu-miR-181a-1-3p	20Gy	1.2626	0.5793	0.8494
mmu-miR-127-3p	20Gy	0.85021	0.5806	0.84957
mmu-miR-484	40Gy	1.134	0.5859	0.85558
mmu-miR-136-5p	80Gy	0.74401	0.5912	0.85924
mmu-miR-19b-3p	40Gy	1.0986	0.5921	0.85924
rno-miR-146b-5p	40Gy	0.78681	0.593	0.85924

Name	Dose	Fold-Change	Permutation Student p-value	Corrected Permutation Student p-value
mmu-miR-802-5p	80Gy	1.2486	0.5932	0.85924
mmu-miR-376a-3p	20Gy	1.1369	0.5975	0.86372
mmu-miR-532-3p	20Gy	0.92961	0.6005	0.86631
rno-miR-322-3p	20Gy	0.66651	0.6062	0.87278
mmu-miR-676-5p	20Gy	0.79029	0.608	0.87362
mmu-miR-150-5p	20Gy	1.0791	0.6094	0.87388
mmu-miR-214-3p	40Gy	1.1158	0.6133	0.87545
mmu-miR-143-3p	20Gy	0.88566	0.6137	0.87545
mmu-miR-337-5p	40Gy	1.2835	0.6152	0.87545
mmu-miR-135a-5p	20Gy	1.1655	0.6162	0.87545
mmu-miR-128-3p	80Gy	0.88575	0.6166	0.87545
mmu-miR-500-3p	40Gy	1.2983	0.6212	0.87952
mmu-miR-148b-3p	80Gy	1.1218	0.623	0.87952
mmu-miR-340-5p	40Gy	0.92931	0.6238	0.87952
mmu-miR-410-3p	20Gy	0.80001	0.6253	0.87952
mmu-miR-184-3p	80Gy	1.5231	0.6272	0.87952
mmu-miR-199b-5p	80Gy	0.77574	0.63	0.87952
mmu-miR-1a-3p	20Gy	0.82337	0.6311	0.87952
mmu-miR-1839-3p	20Gy	0.87407	0.6326	0.87952
mmu-miR-132-3p	80Gy	0.87462	0.6333	0.87952
mmu-miR-7a-5p	20Gy	1.3108	0.6345	0.87952
rno-miR-146b-5p	20Gy	1.1899	0.6349	0.87952
mmu-miR-100-5p	20Gy	0.81602	0.6368	0.87952
mmu-miR-361-5p	40Gy	1.2243	0.6397	0.87952
mmu-miR-411-5p	40Gy	1.3052	0.6401	0.87952
mmu-miR-1930-5p	80Gy	1.2934	0.6412	0.87952
mmu-let-7d-5p	20Gy	1.1231	0.6418	0.87952
mmu-miR-206-3p	40Gy	0.60321	0.6428	0.87952
mmu-miR-28a-5p	40Gy	1.1201	0.6431	0.87952
mmu-miR-429-3p	80Gy	0.81269	0.6434	0.87952
mmu-miR-204-5p	20Gy	0.87506	0.644	0.87952
mmu-miR-101b-3p	40Gy	0.8797	0.6461	0.88013
mmu-miR-328-3p	40Gy	0.89407	0.6469	0.88013
mmu-miR-324-5p	20Gy	0.91089	0.6507	0.88276
mmu-miR-673-3p	80Gy	0.87528	0.6513	0.88276
mmu-miR-495-3p	80Gy	1.1938	0.6534	0.88394
mmu-miR-15b-3p	40Gy	1.1834	0.657	0.88714
mmu-miR-497-5p	80Gy	1.1825	0.6617	0.88895
mmu-miR-296-5p	80Gy	1.2077	0.6619	0.88895
mmu-miR-495-3p	20Gy	0.88401	0.6621	0.88895
mmu-miR-376a-3p	80Gy	1.1587	0.6633	0.88895
mmu-miR-500-3p	20Gy	1.1852	0.6675	0.89291
mmu-miR-15b-3p	20Gy	1.1987	0.6729	0.89798
mmu-miR-497-5p	20Gy	0.88006	0.6738	0.89798
mmu-miR-16-1-3p	40Gy	0.73977	0.6806	0.90536
mmu-miR-31-5p	80Gy	0.90518	0.6847	0.90693
mmu-miR-133b-3p	20Gy	1.2023	0.6868	0.90693
mmu-miR-1897-5p	80Gy	1.1661	0.6869	0.90693
mmu-miR-511-5p	20Gy	0.87752	0.6872	0.90693
rno-miR-532-5p	20Gy	1.0646	0.6897	0.90693
mmu-miR-696	40Gy	0.80207	0.6898	0.90693
U6-snrRNA	40Gy	1.1759	0.691	0.90693
U6-snrRNA	20Gy	1.1851	0.6919	0.90693
mmu-miR-200c-3p-bis	20Gy	1.1675	0.6962	0.9109
mmu-miR-384-5p	40Gy	0.76063	0.6987	0.91251
mmu-miR-335-3p	40Gy	1.1851	0.7038	0.91709
mmu-miR-3107-5p	40Gy	1.1282	0.7072	0.91709
mmu-miR-93-3p	20Gy	1.1016	0.7074	0.91709
mmu-miR-21a-5p	20Gy	0.94982	0.7106	0.91709
mmu-miR-339-5p	20Gy	0.89658	0.7108	0.91709
mmu-miR-497-5p	40Gy	0.78917	0.7113	0.91709
mmu-miR-194-5p	40Gy	0.82096	0.7115	0.91709

Name	Dose	Fold-Change	Permutation Student p-value	Corrected Permutation Student p-value
mmu-miR-421-3p	40Gy	1.113	0.7144	0.91709
mmu-miR-142-5p	40Gy	0.79592	0.7148	0.91709
mmu-miR-335-3p	80Gy	1.1487	0.715	0.91709
mmu-miR-184-3p	40Gy	0.6808	0.7176	0.91791
mmu-miR-26b-3p	80Gy	0.83334	0.7182	0.91791
mmu-miR-9-3p	20Gy	1.1717	0.7198	0.91832
mmu-miR-199a-3p	20Gy	0.94217	0.7242	0.92229
mmu-miR-375-3p	20Gy	0.90712	0.7287	0.92413
mmu-miR-337-5p	80Gy	0.87724	0.7289	0.92413
mmu-miR-15b-5p	40Gy	1.1532	0.7299	0.92413
mmu-miR-30a-5p	20Gy	1.0304	0.7308	0.92413
mmu-miR-1969	80Gy	1.2028	0.7368	0.92884
mmu-miR-26b-3p	40Gy	0.7563	0.7379	0.92884
mmu-miR-141-3p	40Gy	0.90907	0.7392	0.92884
mmu-miR-146b-5p	20Gy	0.94496	0.7397	0.92884
mmu-miR-29c-3p	80Gy	0.94187	0.7431	0.93085
mmu-miR-9-3p	40Gy	1.1483	0.7439	0.93085
mmu-miR-200c-3p	40Gy	1.2463	0.7477	0.93359
mmu-miR-877-3p	20Gy	1.1362	0.7492	0.93359
mmu-miR-92a-3p	80Gy	0.95175	0.75	0.93359
mmu-miR-150-5p	40Gy	0.93396	0.7513	0.93359
rno-miR-7a-1-3p	20Gy	0.92864	0.754	0.93533
rno-miR-143-3p	20Gy	1.249	0.7566	0.93554
mmu-miR-93-3p	40Gy	0.89636	0.7571	0.93554
mmu-miR-132-3p	20Gy	1.0885	0.7597	0.93554
mmu-miR-140-3p	80Gy	1.1639	0.7601	0.93554
mmu-miR-143-3p	40Gy	1.0932	0.7611	0.93554
mmu-miR-128-3p	40Gy	1.0935	0.762	0.93554
mmu-miR-140-3p	40Gy	0.81471	0.7662	0.93877
mmu-miR-221-3p	80Gy	0.8868	0.7682	0.93877
mmu-miR-140-5p	80Gy	0.95954	0.7701	0.93877
mmu-miR-34b-3p	80Gy	0.89668	0.7727	0.93877
mmu-miR-423-5p	80Gy	0.93438	0.7729	0.93877
mmu-miR-378a-5p	40Gy	1.1083	0.7735	0.93877
mmu-miR-18a-3p	20Gy	1.0872	0.7738	0.93877
mmu-miR-875-5p	40Gy	0.78921	0.7781	0.94226
mmu-miR-30a-3p	20Gy	0.90588	0.7793	0.94226
mmu-miR-376c-3p	80Gy	1.0808	0.7811	0.94284
mmu-miR-125b-5p	20Gy	0.92917	0.7845	0.94536
mmu-miR-26a-5p	40Gy	0.94658	0.79	0.94865
mmu-miR-133a-3p	20Gy	0.89273	0.7908	0.94865
mmu-let-7b-5p	20Gy	0.95993	0.7912	0.94865
mmu-miR-324-3p-1	80Gy	1.2564	0.7942	0.95065
mmu-miR-30e-5p	80Gy	0.975	0.7963	0.95156
mmu-miR-215-5p	20Gy	0.94008	0.8005	0.95156
mmu-miR-421-3p	20Gy	0.93232	0.801	0.95156
mmu-miR-30d-5p	20Gy	1.0246	0.8019	0.95156
mmu-miR-199a-3p	80Gy	0.95192	0.8019	0.95156
mmu-miR-1839-3p	40Gy	1.1037	0.8032	0.95156
rno-miR-143-3p	80Gy	0.83809	0.8044	0.95156
mmu-miR-1954	80Gy	0.92024	0.8062	0.95156
rno-miR-148b-5p	40Gy	1.1333	0.8069	0.95156
mmu-miR-92a-3p	40Gy	1.0617	0.8084	0.95176
mmu-miR-1969	20Gy	0.88123	0.8128	0.95289
mmu-miR-34c-3p	20Gy	0.88195	0.8133	0.95289
mmu-miR-27b-3p	40Gy	0.93982	0.8137	0.95289
mmu-miR-200b-3p	40Gy	0.76224	0.8153	0.95289
mmu-miR-101b-3p	80Gy	0.9416	0.8204	0.95289
rno-miR-1-3p	40Gy	1.0998	0.8241	0.95289
mmu-miR-155-5p	40Gy	0.93188	0.8241	0.95289
mmu-miR-324-3p-1	20Gy	1.1693	0.8267	0.95289
mmu-let-7e-5p	40Gy	0.90383	0.8271	0.95289

Name	Dose	Fold-Change	Permutation Student p-value	Corrected Permutation Student p-value
mmu-miR-676-5p	80Gy	0.89996	0.8289	0.95289
mmu-miR-328-3p	20Gy	1.0336	0.8296	0.95289
mmu-miR-200c-3p	20Gy	0.94274	0.83	0.95289
mmu-miR-872-5p	20Gy	0.96397	0.831	0.95289
mmu-miR-223-3p	40Gy	0.94402	0.8314	0.95289
mmu-miR-1a-3p	40Gy	1.0745	0.832	0.95289
mmu-miR-133b-3p	40Gy	1.104	0.8334	0.95289
mmu-miR-384-5p	80Gy	1.1256	0.8353	0.95289
mmu-miR-15a-3p	20Gy	1.0487	0.8384	0.95289
mmu-miR-19b-3p	80Gy	0.97536	0.8404	0.95289
mmu-miR-26a-5p	80Gy	1.0459	0.8412	0.95289
mmu-miR-337-5p	20Gy	1.0655	0.8424	0.95289
mmu-miR-101b-3p	20Gy	0.95037	0.8425	0.95289
mmu-miR-215-5p	40Gy	1.0808	0.843	0.95289
mmu-miR-127-3p	80Gy	1.0726	0.8442	0.95289
mmu-miR-191-5p	40Gy	0.95639	0.8456	0.95289
mmu-miR-31-5p	20Gy	0.95042	0.8459	0.95289
mmu-miR-1896	80Gy	0.90649	0.8464	0.95289
mmu-miR-1930-5p	40Gy	1.1706	0.8466	0.95289
mmu-miR-151-3p	40Gy	0.94168	0.8479	0.95289
mmu-miR-181a-1-3p	40Gy	1.1343	0.8554	0.95768
mmu-miR-18a-5p	80Gy	1.0357	0.8581	0.95768
mmu-miR-342-3p	20Gy	0.96701	0.8592	0.95768
mmu-miR-222-3p	80Gy	0.97361	0.8596	0.95768
mmu-miR-125b-5p	40Gy	1.0516	0.8604	0.95768
mmu-miR-146a-5p	20Gy	0.97985	0.8623	0.95768
mmu-miR-877-3p	40Gy	1.0631	0.8661	0.95768
mmu-miR-135a-5p	80Gy	1.0565	0.8694	0.95768
mmu-miR-376c-3p	20Gy	0.96453	0.8702	0.95768
mmu-miR-484	80Gy	1.0313	0.8708	0.95768
mmu-miR-125b-5p	80Gy	1.0955	0.8719	0.95768
mmu-miR-28a-5p	80Gy	1.0518	0.8726	0.95768
mmu-miR-139-5p	40Gy	0.96936	0.8726	0.95768
mmu-miR-362-3p	20Gy	0.95605	0.8746	0.95768
mmu-miR-203-3p	20Gy	0.97452	0.8757	0.95768
mmu-miR-185-5p	80Gy	1.0278	0.8758	0.95768
mmu-miR-463-3p	40Gy	0.93022	0.8762	0.95768
mmu-miR-145a-5p	40Gy	1.0508	0.879	0.95768
mmu-miR-200c-3p-bis	80Gy	1.0531	0.8791	0.95768
mmu-miR-133a-3p	40Gy	1.0596	0.8807	0.95768
mmu-miR-122-5p	80Gy	0.94317	0.8829	0.95768
mmu-miR-30a-5p	40Gy	0.98643	0.8831	0.95768
rno-miR-224-5p	80Gy	0.94239	0.884	0.95768
mmu-let-7e-5p	20Gy	0.95873	0.8859	0.95768
mmu-miR-463-5p	40Gy	0.90046	0.8887	0.95768
mmu-miR-199b-5p	40Gy	0.89265	0.8891	0.95768
mmu-miR-29b-3p	20Gy	0.93869	0.8916	0.95768
mmu-miR-32-5p	80Gy	0.9799	0.892	0.95768
mmu-miR-376a-3p	40Gy	1.066	0.8934	0.95768
mmu-miR-15a-3p	40Gy	1.0442	0.8937	0.95768
mmu-miR-125a-5p	80Gy	0.97085	0.8937	0.95768
mmu-miR-2183	80Gy	1.0378	0.8949	0.95768
mmu-miR-9-5p	40Gy	1.0536	0.8976	0.95913
mmu-miR-384-5p	20Gy	0.93643	0.8994	0.95963
mmu-miR-148a-3p	20Gy	1.0222	0.9022	0.96118
rno-miR-632	40Gy	1.0773	0.9098	0.96779
mmu-miR-411-5p	20Gy	1.0488	0.9111	0.96779
mmu-miR-1897-5p	20Gy	1.0315	0.9173	0.97207
mmu-miR-362-3p	40Gy	1.0391	0.9179	0.97207
mmu-miR-222-3p	40Gy	0.97923	0.9192	0.97207
mmu-miR-676-3p	80Gy	1.243	0.9244	0.97314
mmu-miR-26b-5p	80Gy	1.0119	0.9267	0.97314

Name	Dose	Fold-Change	Permutation Student p-value	Corrected Permutation Student p-value
mmu-miR-130b-3p	40Gy	0.96768	0.9311	0.97314
mmu-miR-148a-3p	80Gy	0.98372	0.9314	0.97314
mmu-miR-214-3p	80Gy	1.0344	0.9321	0.97314
mmu-miR-181a-5p	80Gy	1.0194	0.9335	0.97314
mmu-miR-674-3p	40Gy	1.045	0.9352	0.97314
mmu-miR-1971	40Gy	1.037	0.9366	0.97314
mmu-miR-143-3p	80Gy	0.98245	0.9394	0.97314
mmu-let-7g-5p	40Gy	0.98554	0.9395	0.97314
mmu-miR-200c-3p-bis	40Gy	1.0325	0.9399	0.97314
mmu-miR-34a-5p	80Gy	0.98531	0.9407	0.97314
mmu-miR-135b-5p	20Gy	0.96251	0.9409	0.97314
U6-snrRNA	80Gy	0.9783	0.9411	0.97314
mmu-miR-674-3p	80Gy	0.97752	0.9429	0.97314
rno-miR-381-3p	20Gy	1.0242	0.9437	0.97314
mmu-miR-191-5p	20Gy	0.98699	0.9451	0.97314
mmu-miR-27a-3p	40Gy	0.98449	0.9452	0.97314
rno-miR-322-3p	40Gy	1.0887	0.946	0.97314
mmu-miR-205-5p	20Gy	0.9826	0.9539	0.97987
mmu-miR-122-5p	40Gy	1.0217	0.9565	0.98113
mmu-miR-350-3p	20Gy	1.012	0.962	0.98536
mmu-miR-194-5p	20Gy	0.98785	0.9651	0.98713
mmu-miR-140-5p	40Gy	1.01	0.9685	0.98781
mmu-miR-200a-3p	20Gy	0.98689	0.9696	0.98781
mmu-miR-434-3p	80Gy	1.0087	0.9699	0.98781
mmu-miR-712-5p	40Gy	1.018	0.9737	0.99027
mmu-miR-379-5p	80Gy	1.0139	0.9768	0.99074
mmu-miR-224-5p	20Gy	0.99368	0.9782	0.99074
mmu-miR-31-5p	40Gy	0.99119	0.9783	0.99074
mmu-miR-676-5p	40Gy	0.99232	0.9822	0.99188
mmu-miR-200b-3p	20Gy	1.0081	0.9822	0.99188
mmu-miR-676-3p	20Gy	0.99669	0.986	0.99432
mmu-miR-7a-1-3p	20Gy	1.0036	0.9896	0.9944
rno-miR-143-3p	40Gy	0.99137	0.9907	0.9944
mmu-miR-712-5p	80Gy	1.0046	0.9934	0.9944
mmu-miR-128-3p	20Gy	0.99858	0.9939	0.9944
mmu-miR-423-5p	40Gy	0.99766	0.9943	0.9944
mmu-let-7c-5p	20Gy	1.0011	0.9944	0.9944

Supplementary Table 3: Univariate analysis of the association of miRNAs with D14 injury scores

Name	Spearman Corr	Spearman p-value	Corrected Spearman p-value
mmu-miR-18a-5p	-0.492	0.006	0.555
mmu-miR-342-3p	0.443	0.007	0.555
mmu-miR-350-3p	-0.441	0.008	0.555
mmu-miR-375-3p	-0.421	0.011	0.555
mmu-miR-511-5p	-0.398	0.02	0.555
rno-miR-345-3p	-0.388	0.021	0.555
mmu-miR-150-5p	-0.382	0.022	0.555
mmu-miR-15b-5p	0.378	0.023	0.555
mmu-miR-146a-5p	-0.376	0.024	0.555
mmu-miR-7a-5p	0.372	0.025	0.555
mmu-miR-223-3p	-0.371	0.026	0.555
mmu-miR-676-3p	-0.367	0.03	0.555
mmu-miR-532-5p	-0.36	0.031	0.555
mmu-miR-215-5p	-0.356	0.033	0.555
mmu-miR-181a-1-3p	0.374	0.035	0.555
mmu-miR-27a-3p	-0.341	0.042	0.625
mmu-miR-200c-3p	0.336	0.045	0.63
mmu-miR-130a-3p	-0.322	0.055	0.705
mmu-miR-29a-5p	-0.32	0.057	0.705
mmu-miR-455-5p	-0.335	0.061	0.705
mmu-let-7i-5p	0.314	0.062	0.705
mmu-miR-139-5p	-0.303	0.073	0.717
mmu-miR-34c-3p	0.302	0.073	0.717
mmu-miR-574-3p	-0.3	0.076	0.717
mmu-miR-192-5p	-0.297	0.079	0.717
mmu-miR-326-3p	-0.308	0.081	0.717
mmu-miR-872-3p	-0.291	0.085	0.717
mmu-miR-463-3p	-0.29	0.086	0.717
mmu-miR-339-5p	-0.297	0.088	0.717
mmu-miR-195a-5p	0.191	0.091	0.717
mmu-miR-24-3p	-0.283	0.095	0.717
mmu-let-7d-5p	0.281	0.097	0.717
mmu-miR-463-5p	-0.278	0.1	0.717
mmu-miR-23b-3p	-0.277	0.102	0.717
mmu-miR-324-3p	-0.271	0.11	0.731
mmu-miR-22-5p	-0.275	0.11	0.731
mmu-miR-126a-5p	-0.267	0.115	0.731
mmu-miR-24-2-5p	-0.27	0.117	0.731
mmu-let-7e-5p	0.261	0.124	0.731
mmu-miR-872-5p	-0.264	0.125	0.731
mmu-miR-411-5p	0.271	0.127	0.731
mmu-miR-15a-3p	-0.258	0.129	0.731
mmu-miR-29a-3p	-0.256	0.132	0.731
mmu-miR-324-5p	-0.257	0.136	0.739
mmu-miR-99a-5p	0.249	0.149	0.777
mmu-miR-22-3p	-0.249	0.155	0.777
mmu-miR-345-5p	0.253	0.155	0.777

Name	Spearman Corr	Spearman p-value	Corrected Spearman p-value
mmu-miR-21a-5p	-0.241	0.156	0.777
mmu-miR-204-5p	-0.238	0.161	0.78
mmu-miR-652-3p	-0.237	0.163	0.78
rno-miR-7a-1-3p	-0.234	0.176	0.799
mmu-miR-696	-0.232	0.187	0.799
mmu-miR-155-5p	-0.225	0.188	0.799
mmu-miR-15a-5p	0.225	0.188	0.799
mmu-miR-27b-3p	-0.225	0.188	0.799
mmu-miR-423-5p	-0.23	0.19	0.799
mmu-miR-1191	-0.235	0.195	0.799
mmu-miR-1894-3p	-0.218	0.2	0.799
mmu-miR-185-5p	-0.218	0.201	0.799
rno-miR-223-3p	-0.221	0.203	0.799
mmu-miR-30a-5p	-0.215	0.208	0.799
mmu-miR-328-3p	-0.214	0.21	0.799
mmu-miR-1839-5p	-0.219	0.213	0.799
mmu-miR-331-3p	0.208	0.224	0.799
mmu-miR-133a-3p	0.207	0.227	0.799
mmu-miR-875-5p	-0.206	0.229	0.799
mmu-miR-142-3p	0.205	0.23	0.799
mmu-miR-664-3p	-0.205	0.238	0.799
mmu-miR-1971	-0.204	0.241	0.799
mmu-miR-30e-3p	-0.2	0.241	0.799
mmu-miR-23a-3p	-0.2	0.242	0.799
mmu-miR-30c-5p	0.199	0.244	0.799
mmu-miR-193a-5p	-0.2	0.249	0.799
mmu-miR-140-5p	-0.196	0.251	0.799
mmu-miR-103-3p	0.195	0.254	0.799
mmu-miR-340-5p	-0.195	0.255	0.799
mmu-miR-500-3p	0.204	0.263	0.799
mmu-miR-374b-5p	-0.191	0.263	0.799
mmu-miR-335-5p	-0.186	0.278	0.799
mmu-miR-186-5p	0.185	0.28	0.799
rno-miR-196c-5p	0.193	0.281	0.799
mmu-miR-1a-3p	0.183	0.285	0.799
mmu-miR-218-5p	0.183	0.285	0.799
mmu-miR-184-3p	0.194	0.288	0.799
rno-miR-1-3p	0.181	0.292	0.799
mmu-miR-1961	-0.18	0.294	0.799
mmu-miR-199b-5p	-0.191	0.296	0.799
mmu-miR-133b-3p	0.178	0.299	0.799
mmu-miR-93-3p	-0.181	0.299	0.799
mmu-miR-1930-5p	0.186	0.301	0.799
mmu-miR-135b-5p	-0.177	0.323	0.849
mmu-miR-194-5p	0.165	0.336	0.849
mmu-miR-190b-5p-bis	-0.166	0.341	0.849
mmu-miR-495-3p	0.17	0.344	0.849
mmu-miR-9-3p	0.166	0.349	0.849
U6-snRNA	0.161	0.349	0.849

Name	Spearman Corr	Spearman p-value	Corrected Spearman p-value
mmu-miR-494-3p	0.167	0.353	0.849
mmu-miR-1897-5p	0.158	0.357	0.849
mmu-miR-126a-3p	-0.157	0.36	0.849
mmu-miR-214-5p	-0.167	0.361	0.849
mmu-miR-877-3p	-0.159	0.363	0.849
mmu-miR-361-5p	-0.163	0.364	0.849
mmu-miR-455-3p	-0.165	0.366	0.849
mmu-miR-29b-3p	0.156	0.37	0.851
mmu-miR-210-3p	-0.152	0.375	0.854
mmu-miR-30a-3p	-0.151	0.381	0.858
mmu-miR-29b-1-5p	-0.151	0.388	0.866
mmu-miR-214-3p	0.143	0.414	0.907
mmu-miR-138-5p	-0.14	0.414	0.907
mmu-miR-338-5p	-0.137	0.424	0.908
mmu-miR-410-3p	0.142	0.424	0.908
mmu-miR-200b-3p	0.137	0.426	0.908
rno-miR-146b-5p	-0.131	0.447	0.921
mmu-miR-301a-3p	0.13	0.45	0.921
mmu-miR-1839-3p	-0.128	0.456	0.921
rno-miR-381-3p	-0.132	0.457	0.921
mmu-miR-149-5p	-0.131	0.46	0.921
mmu-miR-30b-5p	0.123	0.474	0.921
mmu-miR-128-3p	-0.125	0.475	0.921
rno-miR-214-3p	-0.123	0.483	0.921
mmu-miR-191-5p	-0.12	0.485	0.921
mmu-miR-18a-3p	-0.124	0.486	0.921
mmu-miR-1969	0.119	0.497	0.921
mmu-miR-378a-5p	0.116	0.508	0.921
mmu-miR-205-5p	-0.116	0.508	0.921
mmu-miR-467b-3p	-0.114	0.515	0.921
mmu-miR-30d-5p	0.112	0.516	0.921
mmu-miR-30e-5p	-0.112	0.517	0.921
mmu-miR-92a-3p	-0.111	0.518	0.921
mmu-miR-497-5p	0.108	0.531	0.921
mmu-miR-99b-5p	0.108	0.531	0.921
mmu-miR-16-1-3p	-0.111	0.534	0.921
mmu-miR-19a-3p	0.105	0.541	0.921
mmu-miR-425-3p	-0.112	0.541	0.921
mmu-miR-423-3p	-0.105	0.543	0.921
mmu-miR-7a-1-3p	-0.106	0.544	0.921
rno-miR-148b-5p	-0.107	0.546	0.921
mmu-miR-145a-5p	-0.103	0.549	0.921
mmu-miR-193b-3p	0.103	0.549	0.921
mmu-let-7c-5p	0.103	0.55	0.921
mmu-miR-144-3p	0.104	0.55	0.921
rno-miR-532-5p	-0.102	0.558	0.921
mmu-miR-138-1-3p	0.102	0.562	0.921
mmu-miR-181a-5p	0.1	0.563	0.921
mmu-miR-15b-3p	-0.104	0.563	0.921

Name	Spearman Corr	Spearman p-value	Corrected Spearman p-value
mmu-miR-31-3p	-0.097	0.586	0.921
mmu-miR-141-3p	0.097	0.586	0.921
mmu-miR-17-5p	0.094	0.586	0.921
mmu-miR-130b-3p	0.092	0.592	0.921
mmu-miR-200c-3p-bis	0.093	0.593	0.921
mmu-miR-532-3p	-0.092	0.593	0.921
rno-miR-632	-0.092	0.599	0.921
mmu-miR-362-3p	-0.094	0.602	0.921
mmu-miR-146b-5p	-0.088	0.608	0.921
mmu-miR-203-3p	-0.088	0.609	0.921
mmu-miR-337-5p	-0.091	0.61	0.921
mmu-miR-151-3p	-0.088	0.611	0.921
mmu-miR-19b-3p	-0.087	0.613	0.921
mmu-miR-802-5p	0.086	0.616	0.921
mmu-miR-101b-3p	-0.087	0.62	0.921
mmu-let-7g-5p	-0.085	0.621	0.921
mmu-miR-9-5p	0.084	0.628	0.921
mmu-miR-100-5p	0.083	0.635	0.921
mmu-miR-376c-3p	0.081	0.642	0.921
mmu-miR-301b-3p	0.079	0.648	0.921
mmu-miR-200a-3p	-0.078	0.651	0.921
mmu-miR-106a-5p	0.076	0.658	0.921
mmu-miR-673-3p	0.076	0.66	0.921
mmu-miR-101a-3p	0.075	0.662	0.921
mmu-miR-3107-5p	0.075	0.662	0.921
mmu-miR-7b-5p	0.076	0.663	0.921
mmu-miR-712-5p	0.077	0.663	0.921
mmu-miR-376b-5p	-0.076	0.67	0.925
mmu-miR-28a-5p	0.074	0.677	0.925
mmu-miR-484	-0.072	0.677	0.925
mmu-miR-188-5p	-0.071	0.682	0.927
mmu-miR-384-5p	0.072	0.695	0.938
mmu-let-7a-1-3p	-0.067	0.701	0.938
mmu-miR-181c-5p	-0.067	0.703	0.938
mmu-miR-429-3p	0.068	0.709	0.941
mmu-miR-222-3p	-0.061	0.722	0.951
mmu-miR-152-3p	-0.061	0.724	0.951
mmu-miR-148a-3p	0.058	0.738	0.958
mmu-miR-34b-3p	-0.056	0.746	0.958
mmu-miR-148b-3p	-0.055	0.748	0.958
mmu-miR-142-5p	0.055	0.752	0.958
mmu-miR-34a-5p	0.055	0.752	0.958
mmu-miR-451a	0.054	0.753	0.958
mmu-miR-143-3p	0.053	0.761	0.958
mmu-miR-25-3p	0.051	0.766	0.958
mmu-miR-135a-5p	-0.051	0.77	0.958
mmu-miR-1896	-0.05	0.771	0.958
U6-snrRNA	-0.05	0.773	0.958
mmu-miR-335-3p	0.049	0.787	0.964

Name	Spearman Corr	Spearman p-value	Corrected Spearman p-value
mmu-miR-296-5p	-0.046	0.792	0.964
rno-miR-322-3p	-0.046	0.799	0.964
mmu-let-7b-5p	-0.044	0.8	0.964
mmu-miR-140-3p	-0.044	0.8	0.964
mmu-miR-122-5p	-0.043	0.803	0.964
mmu-miR-485-3p	-0.043	0.817	0.964
mmu-miR-20a-5p	0.04	0.817	0.964
mmu-miR-10a-5p	-0.039	0.823	0.964
mmu-miR-29c-3p	0.038	0.825	0.964
mmu-miR-32-5p	-0.038	0.825	0.964
mmu-miR-212-3p	-0.039	0.827	0.964
mmu-miR-379-5p	0.039	0.834	0.967
mmu-miR-1954	-0.035	0.84	0.968
mmu-miR-26a-5p	0.034	0.842	0.968
mmu-miR-199a-3p	0.031	0.857	0.981
mmu-miR-125b-5p	-0.029	0.867	0.986
mmu-miR-365-3p	0.028	0.872	0.986
mmu-miR-31-5p	0.026	0.881	0.986
mmu-miR-674-3p	0.026	0.886	0.986
rno-miR-143-3p	-0.025	0.886	0.986
mmu-miR-322-5p	0.024	0.89	0.986
mmu-miR-324-3p-1	0.025	0.891	0.986
mmu-miR-376a-3p	0.021	0.902	0.993
mmu-miR-93-5p	0.017	0.921	0.993
mmu-miR-26b-5p	-0.017	0.922	0.993
mmu-miR-221-3p	-0.015	0.931	0.993
mmu-miR-106b-3p	-0.015	0.933	0.993
rno-miR-200b-3p	-0.014	0.935	0.993
mmu-miR-2183	0.013	0.942	0.993
mmu-miR-125a-5p	0.012	0.946	0.993
mmu-miR-20b-5p	-0.011	0.948	0.993
mmu-miR-491-5p	-0.011	0.95	0.993
mmu-miR-676-5p	0.01	0.956	0.993
mmu-miR-206-3p	0.009	0.96	0.993
mmu-miR-127-3p	-0.009	0.96	0.993
mmu-miR-16-5p	0.008	0.963	0.993
mmu-miR-136-5p	-0.008	0.964	0.993
mmu-miR-434-3p	-0.008	0.964	0.993
mmu-miR-744-5p	-0.006	0.974	0.997
mmu-miR-26b-3p	0.005	0.977	0.997
mmu-miR-224-5p	0.004	0.981	0.997
rno-miR-224-5p	0.003	0.987	0.997
mmu-miR-421-3p	-0.002	0.989	0.997
mmu-miR-132-3p	-0.001	0.997	0.998
mmu-miR-106b-5p	-0.001	0.998	0.998
